# Promising Drug Candidates and New Strategies for Fighting against the Emerging Superbug *Candida auris*

**DOI:** 10.3390/microorganisms9030634

**Published:** 2021-03-18

**Authors:** Muriel Billamboz, Zeeshan Fatima, Saif Hameed, Samir Jawhara

**Affiliations:** 1 Inserm, CHU Lille, Institut Pasteur Lille, Université Lille, U1167—RID-AGE—Facteurs de Risque et Déterminants Moléculaires des Maladies liées au Vieillissement, F-59000 Lille, France; 2 Junia, Health and Environment, Laboratory of Sustainable Chemistry and Health, F-59000 Lille, France; 3 Amity Institute of Biotechnology, Amity University Haryana, Manesar, Gurugram 122413, India; zfatima@ggn.amity.edu (Z.F.); shameed@ggn.amity.edu (S.H.); 4 UMR 8576-UGSF-Unité de Glycobiologie Structurale et Fonctionnelle, Centre National de la Recherche Scientifique, INSERM U1285, University of Lille, F-59000 Lille, France

**Keywords:** *Candida auris*, antifungal drugs, repurposed drugs, combinatorial therapy, natural compounds, nanoparticles

## Abstract

Invasive fungal infections represent an expanding threat to public health. During the past decade, a paradigm shift of candidiasis from *Candida albicans* to non-albicans *Candida* species has fundamentally increased with the advent of *Candida auris*. *C. auris* was identified in 2009 and is now recognized as an emerging species of concern and underscores the urgent need for novel drug development strategies. In this review, we discuss the genomic epidemiology and the main virulence factors of *C. auris*. We also focus on the different new strategies and results obtained during the past decade in the field of antifungal design against this emerging *C. auris* pathogen yeast, based on a medicinal chemist point of view. Critical analyses of chemical features and physicochemical descriptors will be carried out along with the description of reported strategies.

## 1. Introduction

**Figure 1:**
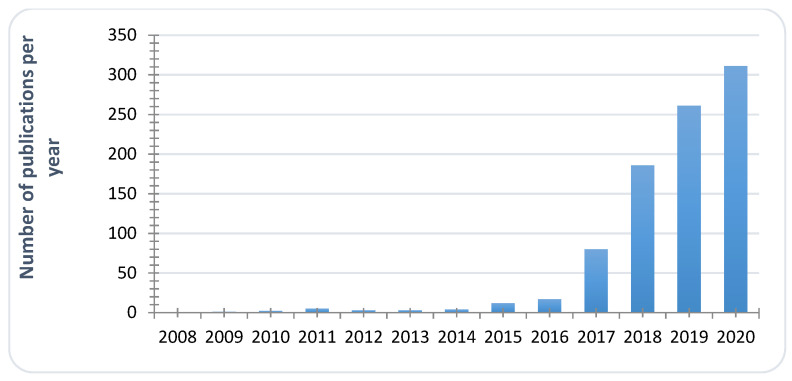
Evolution of annual reports dealing with *C. auris* studies (Scifinder search, key word *Candida auris*, Data Compiled on 31 December 2020).

First identified in 2009 from the ear canal of a patient in Japan [1], *C. auris* is now recognized as an emerging species of concern [2,3], and more and more publications are related to *C. auris* studies (Figure 1). When searching for “*Candida auris*” key words on Scifinder, fewer than 1000 reports have been compiled, most of them during the two last years, proving the acceleration of research and clinical studies around this emerging pathogen. Its emergence is defined by the occurrence of *C. auris* infections in a dozen of countries all around the world. In Europe, since 2015, sporadic epidemics have been reported in Spain [4], United-Kingdom [5], Germany, or Norway [6], for example. Simultaneously, candidiasis caused by *C. auris* were reported in Korea [7], South Korea [8], India [9,10,11], South-Africa [12], or Kowait [13]. In the same period, the United States were also affected [14,15,16]. This human pathogen is associated with severe invasive infections with high mortality rates ranging from 35 to 72% [8,12,17,18]. Since June 2016, governmental institutions (Centers for Disease Control and Prevention (CDC), European Centre for Disease Prevention and Control (ECDC), World Health Organization (WHO), Pan American Health Organization (PAHO), National institute for Communicable Diseases (NICD)…) have issued clinical alerts to health care facilities and provided interim guidelines for clinical management, laboratory testing and infection control of *C. auris* [19,20]. This fungus poses significant challenges to microbiologists and clinicians because of its frequent multidrug resistance; high transmissibility and severe outcomes coupled with misidentification by standard biochemical identification systems such as Vitek 2 [21].

Moreover, the widespread and prolonged use of available antifungal drugs has given rise to multidrug resistance that alters the therapeutic available options. It has been reported that nearly 90% of *C*. *auris* isolates exhibit resistance to fluconazole, around 30% resistance to amphotericin B, and less than 5% resistance to echinocandins [22,23]. Under such compelling circumstances, *C. auris* displays all the features of a “superbug” and efforts are ongoing to identify new therapeutic alternatives to fight against *C. auris* infections. After introducing *C. auris* genomic epidemiology and virulence factors, this review aims at compiling the different strategies and results obtained during the past ten years in the field of antifungal research against emerging *C. auris* from a medicinal chemist point of view. To summarize, six complementary strategies have been developed, from the most attractive to the less reported: (i) repurposing of drugs; (ii) evaluation of combination drugs; (iii) discovery of new drug-candidates; (iv) traditional medicines and natural compounds; (v) metal, metalloids and complexes; (vi) others approaches including nanoparticles and irradiation. Results and data will be analyzed from a chemical approach based on chemical features and physicochemical descriptors such as lipophilicity (expressed by log*P*) and topological polar surface area (TPSA) in order to establish a comparison between classes of compounds and define potential common pharmacophoric moieties. 

## 2. *Candida auris* Genomic Epidemiology and Virulence Factors

### 2.1. Global View

*C. auris* is taxonomically placed as a close relative to the *Candida haemulonii* since its discovery in 2009 from the ear canal of a patient in Japan [1]. *C. auris* was isolated in all continents except Antarctica. Munoz et al. found that four of the five clades of *C. auris* are genetically related to other *Candida* species including *C. haemulonii*, *C. duobushaemulonii*, and *C. psuedohaemulonii* [24]. These five genetically distinct clades of *C. auris* correspond to: Clade I from India and Pakistan (South Asian), Clade II from Japan (East Asian), Clade III from South Africa (African), Clade IV from Venezuela (South American) and most recently a potential Clade V from Iran [22,25]. Over the past ten years, *C. auris* has been isolated across all major continents, including elsewhere in Asia, Africa, North and South America, Australia, Europe, and the Middle East.

Outbreaks in United States have been reported to different clades of *C. auris* that are introduced from other continents. In terms of the analysis of whole-genome sequencing studies, Chow et al. found that the 133 clinical isolates of *C. auris* identified in United-States between 2013 and 2017 were related to South Asian, South American, African, and East Asian isolates but unexpectedly, only 7% of these clinical *C. auris* isolates were identified from patients with clear evidence of being acquired through health-care exposures abroad [16]. Outbreaks in Europe were also attributed to recent spread from other continents. For example, the first case of *C. auris* infection in France has been reported in 2018 from a patient who travelled in India and Iran two months before his hospitalization in France [26].

*C. auris* is believed to have been misidentified as *C. haemulonii* on several occasions, suggesting that *C. auris* has likely been circulating as a human fungal pathogen before 2009. However, retrospective analysis of the SENTRY Antifungal Surveillance collection of over 15,000 *Candida* isolates from 135 participating medical centers in North America, Europe, Latin America, and the Asia-Pacific regions since 1997 shows no misidentifications of *C. auris* before 2009 [27]. 

### 2.2. Virulence Factors of C. auris

**Figure 2:**
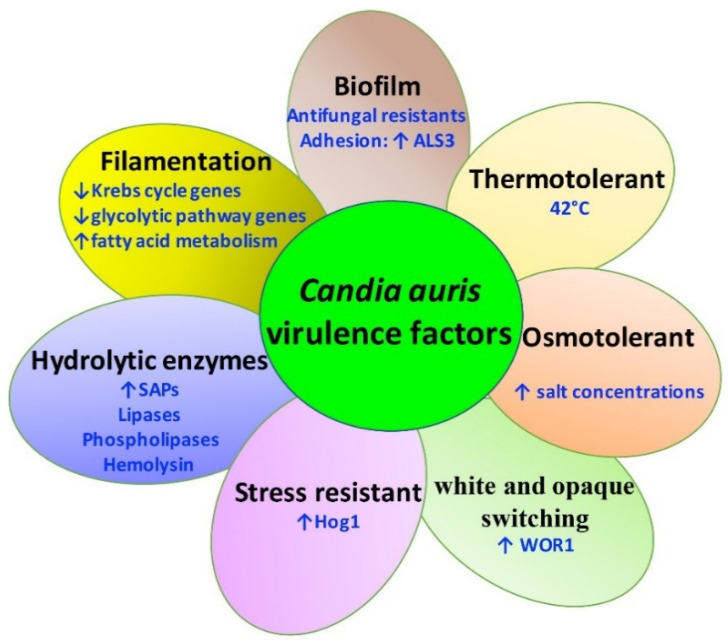
Schematic overview of *C. auris* virulence factors. Stress resistance, hydrolytic enzyme production, thermotolerance, biofilm/adhesion to host cells, osmotolerance, filamentation, and white and opaque switching are important virulence traits in *C. auris.*

Like *C. albicans*, *C. auris* expresses several virulence factors that contribute to pathogenesis including the transition between blastoconidia and filamentous forms, hydrolytic enzyme production, thermotolerance, biofilm/adhesion to host cells, osmotolerance, filamentation, and phenotypic switching (Figure 2).

In contrast to other close relatives including *C. haemulonii* or *C. pseudohaemulonii*, *C. auris* is able to grow at 42 °C [1]. The thermotolerance of *C. auris* contributes to persist against the high fever response and causes an invasive candidemia [28]. Casadevall et al. suggested that the thermotolerance of *C. auris* is related to climate change and global temperature changes supporting the hypothesis that *C. auris* is the first example of a new pathogenic fungus emerging from human-induced global warming [29]. 

*C. auris* is also osmotolerant and known to survive at high salt concentrations, which might enable it to survive environmentally including hypersaline desert lakes, salt-evaporating ponds, or tidal pools (Figure 2) [28]. *C. auris* is able to survive on human skin and environmental surfaces for several weeks and can even tolerate being exposed to some commonly used disinfectants [30]. 

In terms of filamentation of *C. auris*, Yue et al. showed that *C. auris* can switch to three distinct cell types including typical yeast, filamentation-competent (FC) yeast, and a filamentous form [31]. Of note, typical yeast cells are filamentation-incompetent while FC yeast and filamentous cells are filamentation-competent under specific in vitro culture conditions [31]. Furthermore, the difference between the typical yeast and filamentous cell types was variable among the expression levels of metabolism genes. In particular, both Krebs cycle- and glycolytic pathway-associated genes were downregulated in filamentous *C. auris* cells while fatty acid metabolism-related genes were upregulated, supporting that *C. auris* employs different metabolic modes to adapt to its host to reap the overall benefits of its commensal and pathogenic lifestyle [31]. Additionally, some strains of *C. auris* do not produce pseudohypae and show a decreased ability to assimilate different carbon sources (galactose, l−sorbose, cellobiose, l−arabinose, ethanol, glycerol, salicin, or citrate) [1]. 

Another morphological switch implicates the interconversion between white and opaque forms of *C. auris* [32]. Opaque yeast cells are more frequent colonizers in skin infections than white cells, whereas white yeast cells are much virulent in systemic infections than opaque cells [33,34]. Bentz et al. showed that *C. auris* phenotypic switching is regulated by WOR1 that control phenotypic switching in *C. auris* [33].

Like *C. albicans*, *C. auris* can produce the extracellular hydrolytic enzymes that are recognized as an important virulence trait including proteinases, hemolysins, lipases and phospholipases. Regarding secreted aspartyl proteinases (SAPs), that are one of the most significant extracellular enzymes produced in *Candida* species [35], SAPs contribute to degradation of host tissue by providing nutrients for pathogen propagation and promote the inactivation of host antimicrobial peptides, the evasion of the immune responses and the induction of inflammatory mediator release from host cells [36]. In terms of the difference of SAP activity between *C. auris* and *C. albicans*, *C. auris* is able to maintain high SAP activity at 42 °C when compared to that of *C. albicans* supporting that *C. auris* maintains its pathogenicity at higher temperatures [36]. 

Another important group of lytic enzymes are the lipases and phospholipases that are involved in the host damage, immune evasion and biofilm formation [37,38]. In contrast to *C. albicans*, the production of *C. auris* lipases or phospholipases appears to be decreased and strain-dependent although *C. auris* and *C. albicans* share the same quantity of lipase encoding genes in the genome [39]. 

Hemolysin is an exotoxin that is capable of lysing red blood cells as well as nucleated cells and different pathogenic *Candida* species including *C. auris* display hemolysin activity [40]. *C. auris* strains isolated from hospital infections exhibit a high production of hemolysin when compared to those from environmental sources suggesting that hemolysin activity is involved in *C. auris* virulence factors (Figure 2) [41]. 

In terms of stress sensitivities of *C. auris*, Hog1-related stress-activated protein kinase is an important virulence trait for fungal survival against host-imposed stresses and are highly required for the pathogenicity of many fungal pathogens during infection [42]. Day et al. showed that Hog1 is involved in regulating stress resistance, cell morphology, aggregation, and virulence in *C. auris* [43]. 

With regard to biofilm formation, in contrast to *C. albicans* which forms the heterogeneous architecture of biofilms combined with blastoconidia and hyphae embedded within the extracellular matrix, *C. auris* produces thin biofilms composed mostly of blastoconidia and occasionally pseudohyphae embedded within very limited extracellular matrix [40]. Interestingly, these *C. auris* biofilms display lower susceptibility against antifungals including polyenes, azoles, echinocandins and chlorhexidine when compared to those of *C. albicans* suggesting other mechanisms to be more important for this antifungal-resistant biofilm than the reduced biomass of *C. auris* or limited extracellular matrix [43,44,45]. Additionally, adhesion plays a key role in *C. auris* virulence and biofilm formation. Agglutinin-like sequence (ALS) proteins, in particular Als3, are involved in *C. auris* adherence [46]. Singh et al. showed that sera containing anti-Als3 antibodies prevent *C. auris* biofilm formation supporting an important role of Als3 in biofilm formation [47]. Knowing the specificity of *C. auris* and its virulence factors, different strategies are conducted to fight this emerging superbug.

## 3. Repurposing Drugs

### 3.1. Drugs: In Vitro Screening

Repurposing of drugs is an attractive pathway to discover new applications to already developed and filed drugs. Drug repositioning could help spare substantial costs linked to new drug discovery and development. It is also highly interesting because it involves the use of de-risked compounds, avoiding high attrition rate [47,48]. Therefore, this strategy has been applied for repurposing of drugs against *C. auris*. It is notable that the reported studies have been mainly conducted in vitro unless specified. 

**Figure 3:**
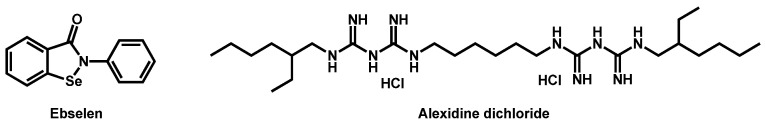
Structures of ebselen and alexidine dichloride.

In that context, the Prestwick Chemical library^®^, a repurposing library of 1280 small mainly off-patent molecules, has been screened several times by different research groups. In 2018, Wall et al. identified, from this library, ebselen as a repositionable molecule [49]. Ebselen is a synthetic organoselenium drug molecule with antioxidant, anti-inflammatory, and cytoprotective activity (Figure 3). It exhibited 100% inhibition of growth of *C. auris* at a concentration of 2.5 µM. In addition, ebselen also inhibited the formation of biofilm and was defined as a broad-spectrum antifungal, not only active on *Candida* species, but also against a variety of medically important fungi, including yeasts and molds. 

Mamouei et al. recently reported the results issued from their own screening of the same chemical collection against *C. auris* [50]. They focused on inhibitors able to block the fungal growth and to kill preformed biofilms. The bis-biguanide alexidine dihydrochloride (AXD) (Figure 3), a potent and selective PTPMT1 (Protein Tyrosine Phosphatase Localized to the Mitochondrion 1) inhibitor, exhibited the highest antifungal and antibiofilm activity against a panel of pathogens, including *C. auris*.

In parallel, from the Prestwick Library^®^, a major work dealing with repurposing of compounds to fight specifically against *C. auris* has been conducted by Zagaroza et al. in 2019 [51]. From this massive screening, 27 drugs proved to inhibit the growth of three different strains of *C. auris* with different geographical origin: CL-10093, JCM 15448, and KCTC 17810 (Table 1). From this pre-selection, the scientists carried complementary studies on 10 drugs, namely MK 801 hydrogen maleate, ciclopirox ethanolamine, trifluoperazine dihydrochloride, suloctidil, ebselen, tamoxifen citrate, rolipram, thiethylperazine dimalate, guanadrel sulfate, and pyrvinium pamoate, and also recently confirmed the described repositionable compounds such as alexidine and ebselen. 

**Figure 4:**
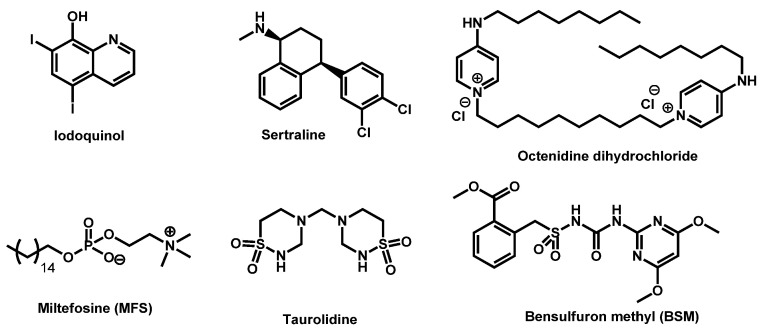
Structures of iodoquinol, miltefosine, sertraline, taurolidine, octenidine dihydrochloride and bensulfuron methyl.

Gowri et al. studied sertraline, a repurposing drug classically used for the treatment of depression, against three different isolates of *C. auris* (Figure 4). Sertraline demonstrated to effectively kill *C. auris* but also to inhibit the formation of biofilm. Its mode of action was elucidated by in silico studies and revealed the binding nature of sertraline to the sterol 14 α-demethylase which is involved in ergosterol biosynthesis [52].

Taurolidine and its derivatives have been recently patented by Diluccio and Reidenberg for the treatment of blood infection by *C. auris* [53]. Taurolidine (Figure 4) is an antimicrobial that is used to try to prevent infections in catheters. 

Octenidine dihydrochloride is a cationic gemini-surfactant, derived from pyridine (Figure 4). More than two decades ago, it was designed for skin, mucous membrane and wound antisepsis. It is currently used as antiseptic in a large field of applications as alternative to chlorhexidine, polyvidone-iodine or triclosan [54]. In 2019, Ponnachan et al. reported the ability of octenidine dihydrochloride to kill *C. auris* strains [55].

Fifty commercially available herbicides, targeting acetohydroxyacid synthase, were recently evaluated by Guddat et al. against *C. auris* CBS10913 strain. Among these compounds, bensulfuron methyl (BSM), belonging to the sulfonylurea chemical subfamily was the most potent discovered antibiofilm formation and antifungal agent (Figure 4) [56].

### 3.2. Drugs: In Vitro Screening and In Vivo Validation

Using medicines for malaria venture’s pathogen box, Wall et al. recently confirmed iodoquinol and miltefosine as potent inhibitors of *C. auris* strain 0390, both under planktonic and biofilm growing conditions (Figure 4) [57]. Both compounds possess broad-spectrum of activity against Candida spp., including multiple strains of the emergent *C. auris*, irrespective of their resistance profiles. Miltefosine (MFS) was also reported in combination with amphotericin B [58]. In the last few months, Barreto et al. confirmed the interest of MFS as an alternative approach to fight against the emerging fungus *C. auris* [59]. They reported its fungicidal activity against planktonic cells of *C. auris* clinical isolates, and antibiofilm ability. They also studied the encapsulation of MFS in alginate nanoparticles (MFS-AN). Using a *Galleria mellonella* larvae infected by *C. auris* model, they demonstrated that both MFS and MFS-AN were able to increase the survival rate. The main advantage of MFS-AN over MFS was its reduced toxicity.

### 3.3. Vaccines: In Vivo Evaluation

Ibrahim et al. reported the efficacy of NDV-3A vaccine to protect mice from multidrug resistant *Candida auris* infection [43,60]. NDV-3A vaccine, harboring the *N*-terminus of Als3p formulated with alum, has been developed initially against *C. albicans.* The authors proved that it generated cross-reactive antibodies against *C. auris* clinical isolates and protected neutropenic mice from *C. auris* infection. Moreover, this repositioning vaccine displayed an additive protective effect in neutropenic mice when combined with micafungin. This vaccine alternative has been recently filed [46].

### 3.4. Critical Analysis of Repurposing Drugs from a Chemical/Physicochemical Point of View

**Figure 5:**
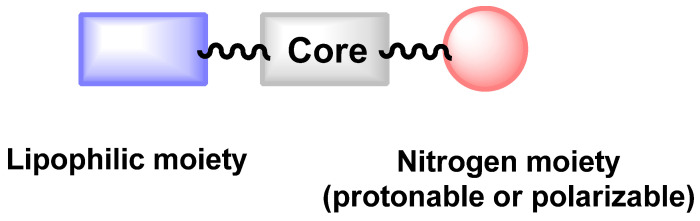
Proposed scaffold of reported *C. auris* antifungals.

From a chemical point of view, it has to be mentioned that the chemical diversity of reported antifungal active against *C. auris* is quite low. Only 30 repositionable compounds have been described for their activity against *C. auris* and most of them can be gathered based on common chemical features: quinoline/isoquinoline, guanidine, phenothiazine/benzothiepine or amide-based cores. These cores are decorated with protonable/polarizable nitrogen groups (pyridine, ternary amine, ammonium, piperazine) and lipophilic moieties (fatty chain or halogenated aromatics). This could be summarized as followed in Figure 5. The protonable nitrogen could be part of the core moiety as in quinoline/isoquinoline derivatives. 

**Figure 6:**
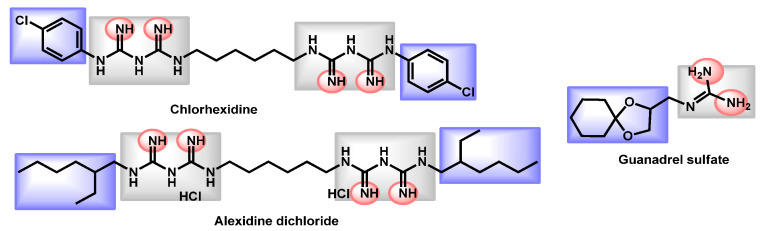
Critical analysis for the guanidine derivatives.

The reported repositionable compounds have been gathered in Table 2, Table 3 and Table 4 and Figure 5 and Figure 6 with analysis of their composition according to the proposed scaffold. 

Concerning the quinoline/isoquinoline sub-group, composed of five molecules, it is notable than the simplest drugs—clioquinol and chloroxine—differed in only one halogen atom. Indeed, the iodine in clioquinol is replaced by a chlorine in chloroxine. This slightly impacted the lipophilicity of the compound as chloroxine is a little more hydrophilic than clioquinol. However, these two compounds could be referred as analogs (Table 2, Entries 1 and 2). In dimethisoquin, the quinoline core is substituted by an additional dimethylamine group which is easily protonable and a fatty butyl chain to enhance the lipophilicity (Table 2, entry 3). Dequalinium dichloride is a bis-quinoline derivative than perfectly fit the proposed scaffold (Table 2, entry 4). In the same manner, pyrvinium pamoate respects the defined features (Table 2, entry 5). On the whole, the quinoline/isoquinoline series is composed of lipophilic compounds (2.86 < log*P* < 4.29). 

Concerning the guanidine derivatives, the same features are also represented (Figure 6). The guanidine core serves also as the protonable part. From a descriptor point of view, these compounds are not blood brain barrier (BBB) permeants. The bis-biguanidines (chlorhexidine and alexidine) are quite lipophilic with log*P* equal to 2.79 and 4.67, respectively. However, guanadrel is quite balanced (log*P* = 0.59). The three drugs exhibited high TPSA (82.86 to 167.58 Å).

Heterocyclic sulfur compounds bearing a phenothiazine or a benzothiepine core displayed good activities against *C. auris*. All are highly absorbed in the gastrointestinal tract and can pass the BBB. The five reported compounds could be divided into two sub-classes and differed only by the nature of the R-substitution on the aromatic moiety (Table 3). Two benzothiepine derivatives (methiothepin and zotepine) were reported. Both are lipophilic (log*P* > 3.9) and exhibited a protonable *N*-methylpiperazine ring as the side-chain. The same *N*-methylpiperazine moiety is kept in the phenothiazine derivatives (prochlorperazine, thiethylperazine, trifluoperazine) with log*P* ranging from 3.47 to 4.53. The introduction of more lipophilic substituents—ethylsulfur and trifluoromethyl—increased the activity against *C. auris* JCM 15448 strain particularly.

The last sub-group which could be addressed is based on an **amide fragment**. The following repositionable drugs have been also analyzed (Table 4). In that case, the polar amide bond could replace the nitrogen-protonable part. However, lipophilic moieties are clearly present, such as *para*-chlorobenzyl or cyclohexyl groups. It is notable that taurolidine, a bis-sulfonamide drug, is the only repositionable drug, which is hydrophilic, with low GI absorption. 

**Figure 7:**
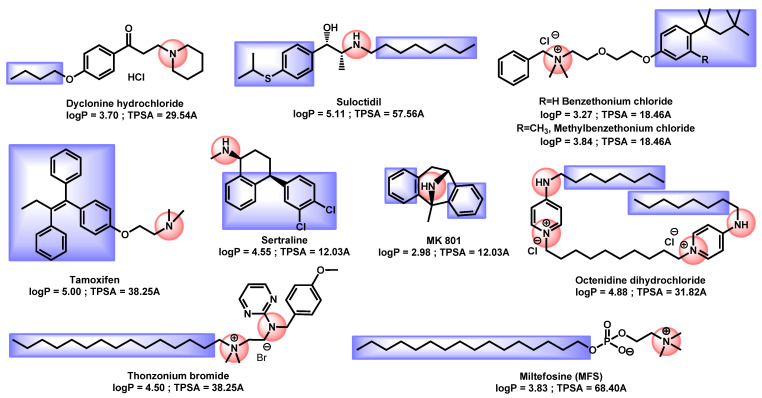
Critical analysis for repurposing drugs.

All others repositionable compounds except artemisin and hexaclophene exhibited a nitrogen protonable group or a quaternary ammonium coupled with a lipophilic moiety even if no common core can be distinguished (Figure 7). Their log*P* ranged from 2.98 to 5.11, proving their lipophilicity. 

## 4. Combination Drugs

Because discovering co-drugs capable of overcoming resistance to frontline antifungals is of prime clinical importance, combination drugs were often reported during the last five years. The potential combinations could be between known antifungal agents (Section 4.1), between repurposing drug and one antifungal (Section 4.2) or between a novel active compound and an “old” antifungal (Section 4.3).

### 4.1. Combination of Antifungal Drugs

To date, only in vitro results are described in this part.

In 2017, Fakhim et al. reported the effects of combination between echinocandins and triazoles against 10 multidrug-resistant *C. auris* isolates [61]. Using a microdilution checkerboard technique, they screened the in vitro interactions of azoles and echinocandins in indian clinical isolates resistant to fluconazole and/or micafungin. They discovered promising synergistic interactions for two couples: micafungin/voriconazole and micafungin/fluconazole. This strategy has also been demonstrated recently by the in vitro synergistic combination of isavuconazole (azole derivative) with colistin [62]. 

In 2020, O’Brien et al. tested if two-drug combinations were effective in vitro against multidrug-resistant *C. auris* isolates [63,64]. From nine reference antifungals and around a thousand tested combinations, they reported that flucytosine (5FC) at 1.0 µg/mL potentiated the most combinations, especially in the case of amphotericin B, echinocandins and voriconazole resistant strains where its addition allowed to restore the fungicidal activity.

### 4.2. Combinations of a Repositioning Drug with an Antifungal Drug

#### 4.2.1. In Vitro Screening

**Figure 8:**
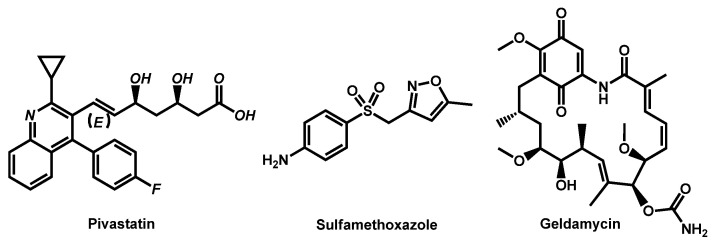
Structure of Pivastatin and Sulfamethoxazole.

In 2018, Seleem et al. studied the combinations between sulfa drugs and reference azole antifungals. Among the active sulfa drugs, the bacteriostatic antibiotic sulfamethoxazole exhibited the most potent in vitro synergistic interactions with voriconazole and itraconazole (Figure 8). The addition of sulfamethoxazole restored the activity of azole drugs against azole-resistant strains if the resistance originated from either overproduction of or decreased affinity for the azole target (ERG11p). Strains resistant because of efflux pump hyperactivity were not susceptible to these combinations [65].

In a parallel manner, in 2020, the same team from Seleem, Mayhoub et al. evaluated the ability of Pharmakon^®^ 1600 drug library to sensitize an azole-resistant *C. albicans* to the effect of fluconazole [66]. They discovered that pitavastatin, a relatively newly developed cholesterol lowering agent, was a potent azole chemosensitizing agent (Figure 8). From a chemical point of view, pivastatin belongs to the quinoline sub-group, including some repurposing drugs. Pivastatin displayed broad-spectrum synergistic interactions with both fluconazole even against *C. auris* strains (MIC from 8 to 64 µg/mL for combination against 64 to 256 µg/mL for drugs alone). Moreover, pitavastatin-fluconazole combination significantly reduced the biofilm-forming abilities of the tested *Candida* species by up to 73%.

In 2019, the team of Chowdhary reported the in vitro effects of combination between geldanamycin (Hsp90-inhibitor) with triazoles and echinocandins against common and emerging *Candida* species (Figure 8) [67]. Whereas synergistic interactions between geldamycin and antifungal drugs were observed for *C. albicans*, *C. glabrata* and *C. parapsilosis*, no interesting effect was observed for any combination against *C. auris*. 

**Figure 9:**
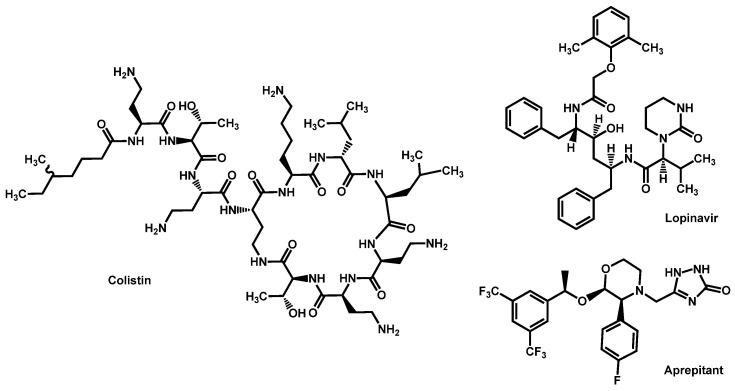
Structure of Colistin, Lopinavir and Aprepitant.

Dannaoui et al. evaluated the in vitro interaction between colistin and two echinocandins (caspofungin and micafungin) against 15 *C. auris* isolates [68]. Colistin, or polymyxin E, is a last-resort antibiotic against multidrug resistant gram-negative infections (Figure 9). It displayed no activity with MIC of >64 μg/mL for all the isolates. However, when colistin was combined with caspofungin, synergistic interactions were observed for all strains.

Very recently, Seleem et al. demonstrated that aprepitant, an antiemetic agent, is a novel broad-spectrum azole chemosensitizing agent (Figure 9). The combination aprepitant/itraconazole is particularly efficient in an in vivo *C. elegans* model of infection by *C. auris*. This synergistic relationship could be mediated by interferences with metal ion homoestasis and subsequent impact on ROS production [69]. The same team also reported that lopinavir, an HIV protease inhibitor, is able to chemosensitize azole drug. 

#### 4.2.2. In Vivo Confirmation

Following the report by Seleem et al., the synergy between sulfamethoxazole and voriconazole was confirmed in vivo in a *C. elegans* model of *C. auris* infection [65].

Moreover, the same group demonstrated in vivo that the lopinavir/itraconazole combination decreased the fungal burden in *C. auris*-infected *C. elegans* nematodes by 88.5% and enhanced their survival rate by 90% relative to that of the untreated control [70]. Comparing lopinavir and aprepitant, both studies reported by the same team on the same in vivo protocols, lopinavir seems to be more potent than aprepitant in the optimized combination with itraconazole. 

### 4.3. Combination of New Compounds and Old Drug: In Vitro Results

**Figure 10:**
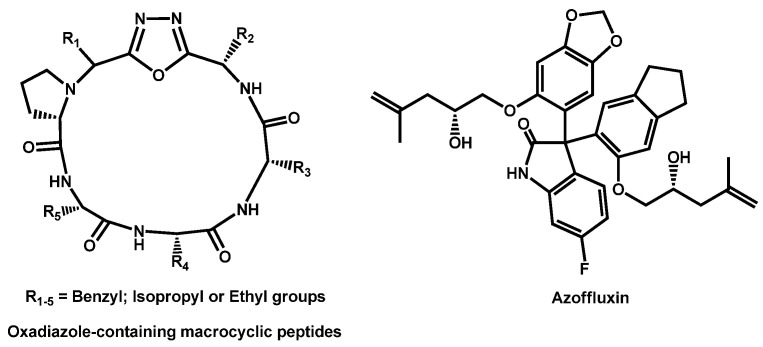
Oxadiazole-containing macrocylic peptides and azoffluxin.

Revie, Robbins et al. recently described the ability of oxadiazolecontaining macrocyclic peptides to potentiate the antifungal activity of fluconazole [71]. Few of the newly obtained oxadiazole-containing macrocyclic peptides displayed activity against Candida spp on their own, but many increased the efficacy of fluconazole, resulting in a synergistic combination (Figure 10).

The synthetic library created by Boston University’s Center for Molecular Discovery^®^ (BU-CMD) was screened at 50 µM to identify azole-synergizing compound. Azoffluxin, due to its strong synergistic interaction with fluconazole against a resistant strain of *C. auris* Ci6684 was identified (Figure 10). Azoffluxin enhances azole efficacy in a Cdr1-dependent manner [72].

**Figure 11:**
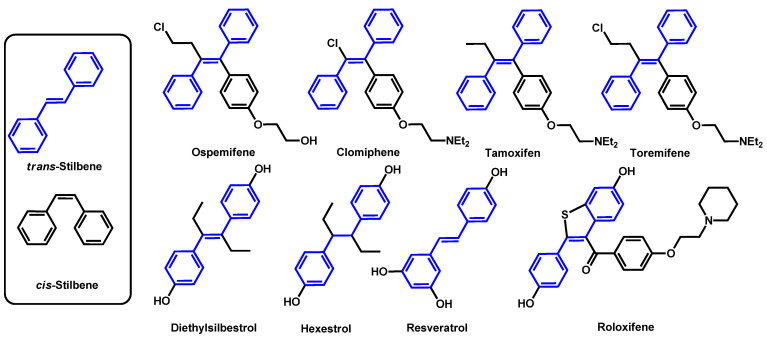
Stilbene compounds tested by Seelem et al. in combination with itraconazole.

Seleem et al. tested nine stilbene compounds for their ability to interact synergistically with azole drugs, particularly against azole-resistant fungal isolates (Figure 11) [73]. Ospemifene displayed the most potent azole chemosensitizing activity and increased the potency of itraconazole against *C. auris*. Indeed, when MIC are superior to 256 µg/mL for both ospemifene and itraconazole alone, their combination exhibited a MIC equal to 2 µg/mL. Moreover, the authors determined that ospemifene interfered directly with fungal efflux systems, thus allowing the fungal intake of itraconazole.

Ahmad et al. examined the effects of combination of natural monoterpene phenols with antifungal drugs against 25 clinical isolates of *C. auris* [74]. None of the monoterpene phenols was active alone against *C. auris* (Table 5). Carvacrol was the most active phenol with median MIC of 125 µg/mL and its combination with amphotericin B, nystatin, fluconazole and caspofungin resulted synergistic and additive effects in 64%, 96%, 68%, and 28%, respectively.

## 5. Novel Compounds

In this section, we reviewed series of novel compounds which have displayed a good to potent activity against *C. auris*. For each chemical class, a critical analysis based on its chemicophysical descriptors will be performed for comparison purpose. The first section is devoted to compounds with potent in vitro activities, whereas the second section concerns chemical classes with proved in vivo efficacy.

### 5.1. Small Molecules

#### 5.1.1. In Vitro Evaluation

**Figure 12:**
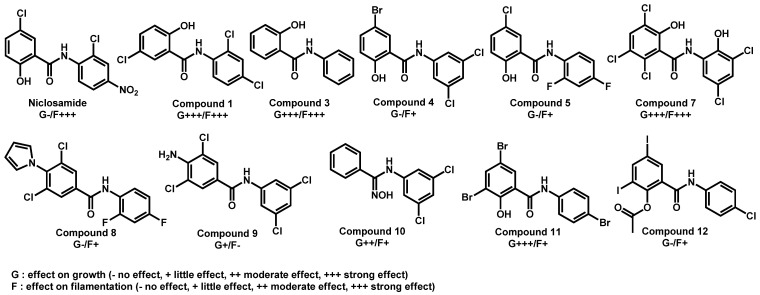
Series of derivatives from niclosamide and their activities towards growth and filamentation as reported by Selam et al.

In 2018, in order to target virulence traits, Selam et al. described the screening of a 698 small-molecule collection against the invasive hyphal growth of *C. albicans* [75]. From their data, niclosamide, an FDA-approved anthelmintic in humans and one of its analogue (*N*1-(3,5-dichlorophenyl)-5-chloro-2-hydroxybenzamide), an halogenated salicylanilide (**1**)—emerged as capable of inhibit both filamentation and biofilm formation. Based on these preliminary results, they extended their work to a series of a dozen of compounds to improve their biological activities. Therefore, they brought to light compounds **3** and **7**, which displayed both anti-filamentation and biofilm formation capacities (Figure 12). This series of compounds are amide-based small molecules with balanced lipophilicity induced by addition of halogenated substituents. They could be related to the previously described amide-based repurposing drugs. As example, log*P* of Compound **1** was calculated at 4.10 and its topological polar surface area equal to 49.33Å, which is in accordance with the values observed for repurposing drugs active against *C. auris*. 

**Figure 13:**
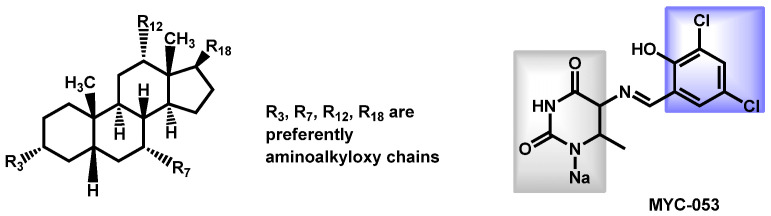
CSA steroids as patented by Genberg, Beus and Savage and MYC-053 as reported by Tetz.

In 2018, Genberg et al. also filed a patent concerning methods for treating fungal infections using a cationic steroid antimicrobial (CSA) [76]. Some cationic steroid antimicrobials have been described and exhibited a broad-spectrum of activity against a panel of fungi, including Candida spp. (Figure 13). In such structures, the steroid core is highly lipophilic and the aminoalkyloxychains could bring a little hydrophilicity and also protonable primary or secondary amino groups.

In 2019, Tetz et al. described the in vitro activity of a novel compound named MYC-053 (Figure 13) against clinically significant multidrug resistant strains such as *C. glabrata*, *C. auris*, *C. neoformans* and Pneumocystis spp. [77,78]. Under their screening conditions, MYC-053 proved to be equally effective against both susceptible control strains, clinical isolates, or resistant strains and exhibited MICs ranging from 0.125 to 4.0 µg/mL. The profile of activity of MYC-053 is unique, because, unlike other antifungals such as azoles, polyenes, and echinocandins, MYC-053 was effective against *Pneumocystis* isolates. The compound is currently under development by TGV-Therapeutics. MYC-053, as a sodium salt, is hydrophilic (log*P* = −0.60). Its topological polar surface area is equal to 78.76 Å. The pyrimidine dione core (in grey) is quite similar to amide-based derivatives and the 2-hydroxy-3,5-dichlorophenyl moiety helped to increase the lipophilicity. 

**Figure 14:**
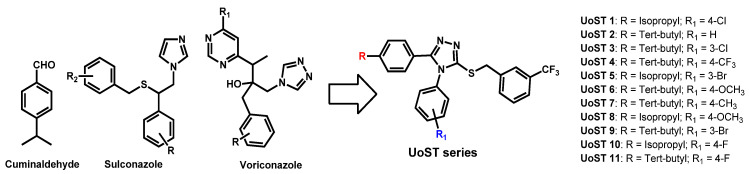
Rational design of cuminaldehyde-azole hybrid compounds by Soliman et al.

In 2020, Soliman et al. described the chemical association of the essential oil cuminaldehyde and azole moieties (as present in sulconazole or voriconazole) in order to take advantage of their individual previously reported anti-candidal properties (Figure 14) [79]. UoST series was evaluated against *C. albicans* and *C. auris*. Only UoST5, UoST7, UoST8 and UoST11 displayed good activity against *C. auris* (MIC ranging from 2 to 15 µg/mL). Obtained by a six-steps pathway, UoST5, their best designed hybrid molecule exhibited a 2 µg/mL MIC_50_ against *C. auris* where amphotericin B displayed MIC_50_ = 0.3 µg/mL. Compound UoST5 was then formulated into PLGA nanoparticles (NPs) and this formulation proved to enhance and prolong the anti-candidal activities of UoST5. OsST5 is a very lipopholic triazole-based compound (log*P* = 7.11) with moderate polar surface area (TPSA = 56.01). 

**Figure 15:**
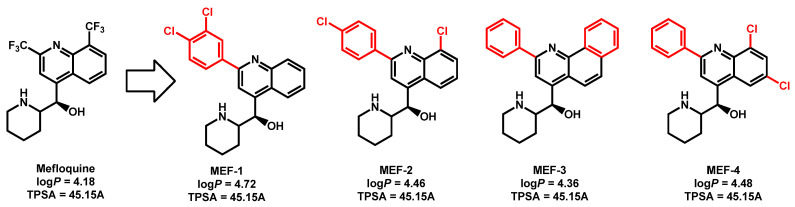
Structure of the derivatives of mefloquine as reported by Montoya et al.

Montoya et al. reported that derivatives of the anti-malarial drug mefloquine have broad-spectrum antifungal activity against pathogenic yeasts and molds [80]. These derivatives were tested against a panel of fungal strains, including *C. auris* strain 0381 and they revealed MIC from 2–4 µg/mL compared to MIC = 128 µg/mL for mefloquine itself (Figure 15). From a chemical point of view, mefloquine and its derivatives belongs to the quinoline chemical class. They displayed the three features defined previously: (i) quinoline core; (ii) lipophilic moiety (halogenated aromatics) and (iii) piperidine protonable nitrogen group. Mefloquine derivatives are quite lipophilic (log*P* from 4.36 to 4.72) with moderate TPSA. 

Orofino et al. reported in 2020 the potency of BM1, a macrocyclic amidinourea active against azole-resistant *Candida* strains [81]. The authors conducted a complete characterization of the in vitro and in vivo biological properties of BM1 and established its good ADME and biochemical characteristics so as its high activity towards several *Candida* species, including preliminary data against *C. auris* (Table 6). BM1 exhibited a log*P* of 3.38 and a very high topological polar surface area of 116.13 Å. From a chemical point of view, it belongs to the guanidine derivatives.

**Figure 16:**
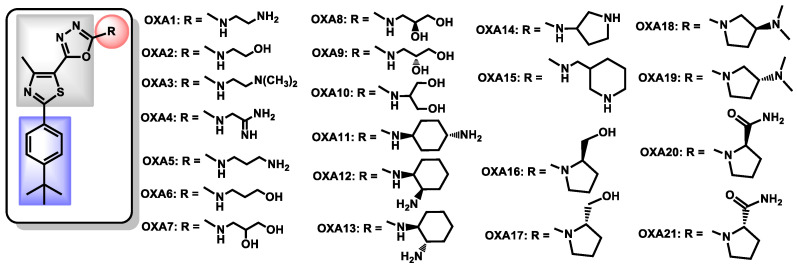
Structures of OXA compounds described by Seleem, Mayhoub et al.

Seleem, Mayhoub et al. are widely involved in the development of polysubstituted thiazoles as novel antibacterial agents. Based on structure-activity relationships studies, they recently described oxadiazolylthiazoles as novel and selective antifungal agents [82]. Via a five-steps pathway, the authors obtained a series of oxadiazolylthiazole OXA1-21 (Figure 16) and evaluated them against a panel of fungal and bacterial strains. OXA11 proved to be selective towards fungal strains and the most active compound from this series, with MIC = 2–4 µg/mL against *C. auris* strains. Moreover, OXA11 exhibited a fungistatic behavior, a broad-spectrum activity against fungal pathogens, with capability of disrupting biofilms but without harming the human normal microbiota. The OXA derivatives perfectly respect the three common features: (i) an original oxadiazolylthiazole core (in grey) coupled with (ii) a lipophilic moiety (in blue) and (iii) a protonable amine group (cyclic or acyclic). OXA11, the most potent reported molecule from this series, displayed log*P* equal to 4.44, a high topological surface area of 118.10 Å and no alert concerning its drug likeliness. 

**Figure 17:**
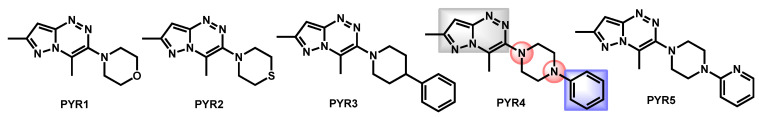
Series of pyrazolo[5,1-c][1,2,4]triazines described by Seleem et al.

Seleem et al. also described the synthesis and biological evaluation of a small series of new pyrazolo[5,1-*c*][1,2,4]triazines against a panel of fungal strains, including *C. auris* (Figure 17) [83]. Compound PYR4 was the most potent antifungal, with a moderate MIC around 15–20 µg/mL. The chemical feature of PYR4 is composed of a pyrazolotriazine core, a piperazine protonable nitrogen group and a lipophilic aromatic (see Figure 17). PYR4, the most potent reported molecule from this series, displayed log*P* equal to 2.16, a medium topological surface area of 49.56 Å and no alert concerning its drug likeliness. However, compared to other series developed by Seleem et al., PYR derivatives are less powerful than OXA or PHE series.

**Scheme 1:**
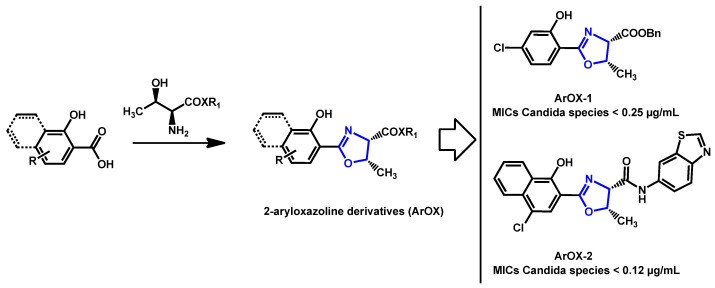
Synthesis of 2-aryloxazoline derivatives (ArOx) and the two most potent compounds ArOX-1 and ArOx-2.

Late 2020, Ishida and Stephani reported 2-aryloxazoline compounds, obtained from L-threonine and aromatic carboxylic acid as promising antifungal compounds (Scheme 1) [84]. They discovered ArOX-1 and ArOX-2, which exhibited a strong in vitro antifungal activity, with MIC lower than 0.25 µg/mL for all tested *Candida* species. Moreover, these two non-toxic compounds were particularly active against *C. auris* CBS10913 (MICs = 0.06 µg/mL) and CBS12766 (MICs = 2 µg/mL).

**Scheme 2:**
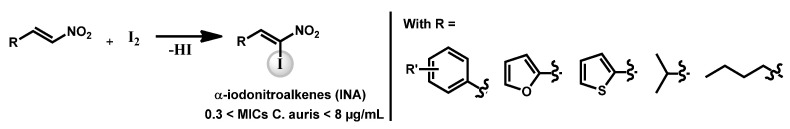
α-Iodonitroalkenes synthesis.

Ganesh et al. discovered a series of α-iodonitroalkenes as potential antifungal and antitubercular compounds. Based on a α-iodation on β-nitroalkenes, the authors obtained a series of derivatives (Scheme 2) which exhibited interesting antifungal activity against *C. auris* with MIC lower than 8 µg/mL for all compounds. It is notable than the furan and thiophene derivatives were the most broad-spectrum antifungals devoted of cytotoxicity [85].

**Scheme 3:**
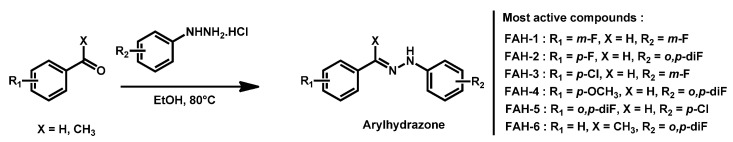
Series of fluorinated hydrazone derivatives (FAH) as reported by Watt and Garneau-Tsodikova.

Among 40 tested hydrazone derivatives, fluorinated aryl- and heteroaryl-substituted hydrazone (FAH) have demonstrated broad-sprectrum fungicidal activities, antibiofilm capacity coupled with low cytotoxicity and did not trigger the development of resistance when exposed to *C. auris* (Scheme 3) [86]. Compounds FAH-1 to FAH-6 exhibited MIC < 1 µg/mL against nine of the 10 different *C. auris* strains. From a chemical point of view, these compounds fit the previously described model for *C. auris* antifungals.

#### 5.1.2. In Vitro Screening and In Vivo Validation

First described in 2008 by Mitsuyama et al. [87], T-2307, an arylamidine, exhibited potent broad-spectrum activities against the majority of fungal pathogens [88], even against echinocandin-resistant *C. albicans* and *C. glabrata* strains [89,90]. T-2307 is transported into *C. albicans* by a high-affinity spermine and spermidine carrier regulated by Agp2 [91], and causes collapse of mitochondrial membrane potential in yeast [92]. In 2020, Patterson et al. reported that arylamidine T-2307 as very active in vitro and in vivo antifungal against *C. auris* (Figure 12) [93]. In vitro MIC ranged from 0.125 to 4 μg/mL at 100% inhibition. Moreover, 3  mg/kg subcutaneous once daily treatment with T-2307 revealed an improved survival rate and reduced kidney fungal burden compared to control. In 2019, T-2307 was acquired by Appili Therapeutics Inc. for development and is now called ATI-2307.

**Figure 18:**
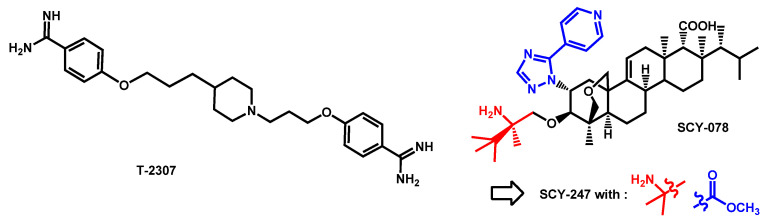
Structure of T-2307 and SCY-078 as reported by Patterson et al. and Larkin et al., respectively. Structure evolution of second generation fungerp SCY-247.

From a chemical point of view, T-2307 displayed the features defined in the previous Figure 18. It exhibited two benzimidamide groups which are linked by a lipophilic propyl chain to a protonable *N*-subtituted piperidine. From this structure, compound T-2307 is rather like the guanidine sub-group.

In 2017, Larkin et al. reported the effects of the orally available SCY-078, a novel 1,3-β-D-glucan synthesis inhibitor, on growth morphology and biofilm formation in *C. auris* (Figure 18) [40]. SCY-078 is a triterpene compound which differs from echinocandins by its oral bioavailability and its non-sensitivity towards the most common mutations within the protein target *Fks*. SCY-078 exhibited an MIC_90_ of 1 mg/liter against *C. auris* by interrupting cell-division. Additionally, SCY-078 displayed powerful antibiofilm activity, by reducing metabolic activity and thickness compared to the untreated control. In parallel, Berkow et al. demonstrated the in vitro activity of SCY-078 on a collection of 100 isolates, representative of the four clades of *C. auris*. The overall mode of SCY-078 was 1 µg/mL with MIC_50_ and MIC_90_ equal to 0.5 µg/mL and 1 µg/mL, respectively. SCY-078 also showed very little variation in activity between the clades compared to micafungin, caspofungin or anidulafungin [94]. Due to its first encouraging data, SCY-078 was referred as a promising new compound by McCarthy and Walsh in their review about new strategies and challenges against emerging and resistant fungal pathogens [95]. Since its discovery, SCY-078 (named Ibrexafungerp) has been integrated in a dozen of clinical trials studies and patented [96]. Concerning *C. auris*, one particular multicenter, open-label, non-comparator, single-arm study to evaluate the efficacy, safety, tolerability and pharmacokinetics of oral SCY-078 as an emergency use treatment for patients with a documented *C. auris* infection is still ongoing (ClinicalTrials.gov Identifier: NCT03363841). Recently, in 2020, Arendrup et al. investigated the in vitro activity of SCY-078 against *C. auris* by applying EUCAST methodology and compared its potential against *C. albicans* and *C. glabrata* and in reference with six control drugs (anidulafungin, micafungin, amphotericin B, fluconazole, voriconazole, and isavuconazole) [97]. More than 150 strains with various resistance profile were screened and proved to be uniformly susceptible to SCY-078. In parallel, Chaturvedi et al. reported that Ibrexafungerp was efficient against pan-resistant (defined as in vitro resistance to two or more azoles, all echinocandins and amphotericin B) *C. auris* isolates from the outbreak in New-York [98] and Ghannoum et al. demonstrated that it was active to control skin infection and colonization of hospitalized patients [99]. Recently, a review compiled data on Ibrexafungerp, established it is a promising, new antifungal agent to treat *C. auris* infections, as patients experienced a complete response after treatment [100].

From a chemical point of view, SCY-078 is based on a lipophilic tetracyclic-fused core with addition of protonable nitrogen moieties (pyridine, triazole and primary amine). Its structure is in accordance with the common defined scaffold.

SCY-247, a second-generation fungerp which differs from SCY-078 by two chemical moieties (the 4-(1H-1,2,4-triazol-5-yl)pyridine and 2,3,3-trimethylbutan-2-amine moieties in SCY-078 that have been replaced by a methylester and a 2-methylpropan-2-amine in SCY-247, respectively (Figure 18) has been recently reported by Ghannoum et al. and proved similar activities against a panel of *Candida* strains and enhanced potency against *C. auris* strains. Moreover, SCY-247 retained its potential even when pH decreased [101].

**Scheme 4:**
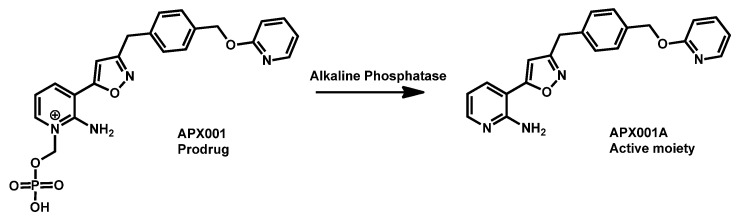
Structure of prodrug APX001 and active moiety APX001A.

First reported in 2018, APX001A (formely E1210, Amplyx) is an antifungal agent that inhibits the fungal enzyme Gwt1 in the glycosylphosphatidylinositol (GPI) biosynthesis pathway. APX001 is the prodrug of APX001A (Scheme 4). Both APX001A and APX001 have been shown to be effective against a variety of fungal species, including Candida spp., Aspergillus spp. and other filamentous fungi. Compound APX001 has emerged as a novel potent antifungal agent against *C. auris*. It has been successfully tested against 100 geographically distinct clinical isolates and exhibited high MIC_50_ and MIC_90_ in the nanogram per liter range [102,103]. Extended evaluation of large panels of strains from different origins has confirmed the interest of APX001 in the treatment of infection by *C. auris* [104]. The compound APX001 has entered clinical trials and proved good efficacy in mice model of candidiasis [105,106]. Fosmanogepix (APX001) is currently in Phase II trials for invasive fungal infections. Both fosmanogepix and manogepix (APX001A) are currently under investigation and proofs of their in vivo efficacy against *C. auris* infection accumulate [105,106,107,108,109]. When analyzing its chemical structure, it appeared that APX001A displayed protonable nitrogen moieties (primary amine and pyridine) coupled with a lipophilic benzyl group. This balance gave a log*P* equal to 3.21 for compound APX001A with TPSA of 87.06 Å which is in the range observed for repurposing drugs. Its prodrug is much more polar (TPSA of 176.84 Å) and hydrophilic (log*P* = 1.70).

**Scheme 5:**
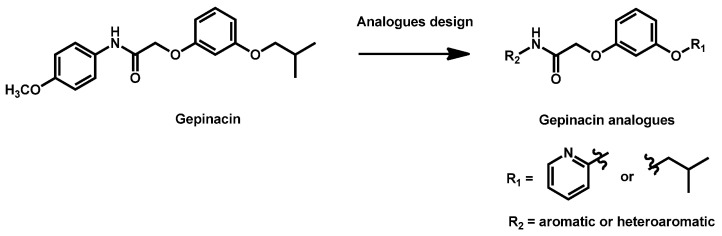
Structure of gepinacin and modulations.

Gepinacin is a pre-clinical Gwt-1 inhibitor which is structurally different from manogepix. Despite high in vitro antifungal activity, gepinacin failed in in vivo trials due to its low metabolic and serum stability. A series of analogues was then designed and evaluated by Walsh and Cowen in 2020 (Scheme 5) [110]. A dozen of compounds have been obtained and exhibited MIC in the range 0.625–10 µg/mL against *C. auris*. However, despite interesting potency and promising single-dose pharmacokinetics, the identified lead (R_1_ = isobutyl; R_2_ = 4-chlorophenyl) did not reveal any activity at the maximal dose in a neutropenic rabbit model of disseminated candidiasis.

**Figure 19:**
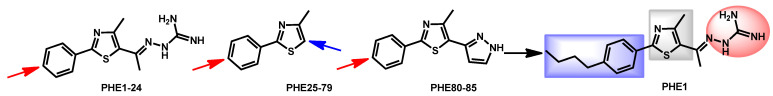
Modifications around the phenylthiazole compounds as reported by Seleem et al.

In 2019, the group headed by Seleem discovered that phenylthiazole small molecules possess dual antifungal and anti-biofilm activities against *C. albicans* and *C. auris* [111]. They tested 85 synthetic phenylthiazole derivatives (Figure 19) and compound PHE1 emerged as the most potent molecule, with MIC ranging from 0.25 to 2 µg/mL against a panel of *C. albicans* and *C. auris* strains. The HIT compound also exhibited anti-biofilm ability, fungicidal activity and ability to prolong survival in infected *Caenorhabditis elegans* model. From a chemical point of view, PHE1 perfectly fit the previously defined scaffold with a hydrazinecarboximidamide moiety (in red) which is a guanidine isostere, a thiazole core (in grey) and a 4-butylphenyl lipophilic chain (in blue). PHE1 displayed a log*P* = 3.64 and TPSA = 115.39 Å which are in accordance with the other reported drugs.

**Figure 20:**
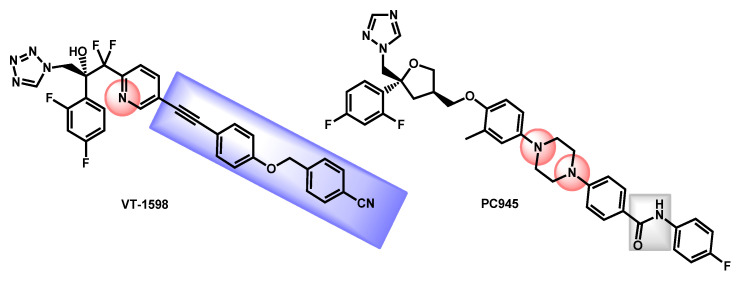
Structures of VT-1598 and PC945.

The work by Wiederhold et al. described the evaluation of VT-1598, a fungal CYP51-specific inhibitor against *C. auris* infections [112]. The tetrazole VT-1598, developed by Viamet Pharmaceuticals (Figure 20) has demonstrated in vitro activity against various fungi [113,114] and has also shown promising results in experimental models of invasive fungal infections such as central nervous system coccidioidomycosis or cryptococcal meningitis [115,116]. The authors demonstrated the in vitro and in vivo efficacy of VT-1598 against *C. auris* infections. VT-1598 is currently on Phase I clinical trials for coccidioidomycosis in order to evaluate its safety and PK of single oral doses in healthy patients (ClinicalTrials.gov Identifier: NCT04208321). From a chemical point of view, VT-1598 is a tetrazole-based compound which exhibited a protonable pyridine link and a lipophilic aromatic chain (in blue). Its lipophilicity (log*P* = 5.12) and polar surface (TPSA = 109.74 Å) are quite high and its gastrointestinal absorption is estimated as low.

Still in 2019, Rudramurthy et al. described the activity of a novel topical triazole named PC945 against *C. auris* (Figure 20) [117]. PC945, optimized for topical application, proved to be 7.4-fold and 1.5-fold more potent than voriconazole and posaconazole against a collection of more than 50 clinical isolates from India, UK, USA, Japan and South-Korea. Three isolates were found to be cross-resistant to PC945 and other azoles but with no clear evidence of involved mutations. The tolerability, efficacy, and safety of PC945 were evaluated by four clinical trials in the frame of aspergillosis and candidiasis recently (ClinicalTrials.gov Identifier: NCT03870841; NCT03905447; NCT03745196 and NCT02715570). Three studies have been interrupted because of the COVID19 outbreak and results are not available. From a chemical point of view, PC945 exhibited a triazole ring—present in several antifungal references’ compounds—and an amide link. The integration of a piperazine link resulted in easily protonable nitrogen groups and fluorinated aromatics increased the lipophilicity. Its lipophilicity (log*P* = 5.70) and polar surface (TPSA = 84.75 Å) are quite high and its gastrointestinal absorption is estimated as low. These values and the presence of a 2,4-difluorophenyl moiety gave PC945 some similarities with VT-1598.

Overall, a dozen novel antifungal chemical families have been reported during the last few years and some of the lead products are currently under clinical trials investigations. From a chemical point of view, these compounds exhibited the same features as discovered repurposing compounds, divided into three complementary parts: (i) core; (ii) protonable nitrogen groups; (iii) lipophilic moieties (generally halogenated aromatics or fatty chains).

### 5.2. Echinocandins

**Figure 21:**
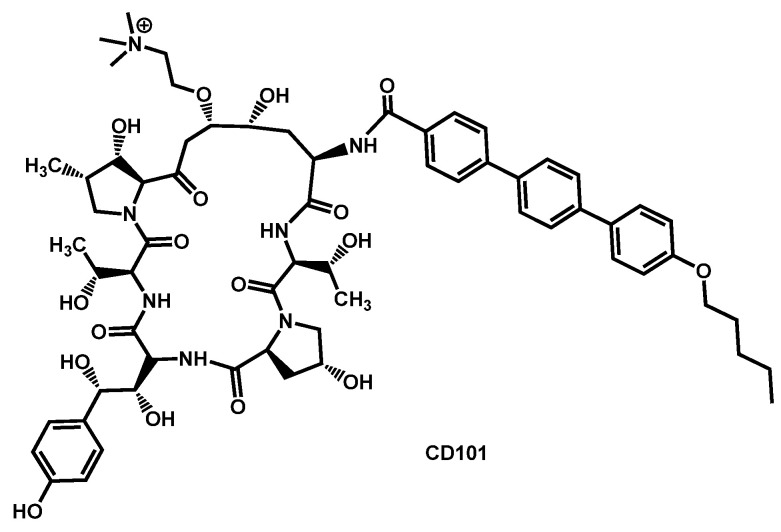
Structure of CD101.

In 2017, CD101 (then named rezafungin) was patented for the treatment of fungal infections (Figure 21) [118]. CD101 is a novel echinocandin with long-acting profile (Figure 21). In 2018, another patent was filed for the use of CD101 alone or in combination with another antifungal drug for the treatment of fungal infections [119]. Overall, CD101 shows encouraging activity against the emerging pathogen *C. auris*. In late 2017, Berkow and Lockhart reported the activity of CD101 against 100 clinical isolates of *C. auris* [120].

They observed that the MICs values ranged from 0.03 to 8 µg/mL, the average MIC_90_ was 0.5 µg/mL. In particular, CD101 was investigated against *C. auris* strains resistant to other echinocandins. The results showed a better activity of CD101 compared to other echinocandins, excepted for isolates containing the S639P amino acid substitution in *FKS1* hot spot 1 which is related to previously reported mutations linked to echinocandin resistance in other Candida spp. A few months later, Hager et al. examinated the efficacy of rezafungin (previously CD101) in the treatment of disseminated *C. auris* infection using an immunocompromised mouse model [121]. Rezafungin proved to have significantly lower average CFU/g of kidney tissue compared with amphotericin B, micafungin and vehicle after ten days of treatment. The pharmacodynamics of rezafungin was then evaluated by Lepak et al. in a neutropenic mouse invasive candidiasis model of *C. auris* infection [122]. In 2019, Majoros et al. extended the study on CD101 and determined the in vitro susceptibility of 689 clinical isolates of 5 common and 19 rare *Candida* species, as well as *Saccharomyces cerevisiae* [123]. Their results proved that rezafungin was an excellent antifungal against common and non-common *Candida* species, with results similar to other echinocandins excepting caspofungin. In 2020, Helleberg et al. investigated the potency of rezafungin and comparators against 1293 Nordic yeast isolates and 122 Indian *C. auris* isolates proving that rezafungin had species-specific activity comparable to that of micafungin and anidulafungin. Even if rezafungin was highly active against the majority of common *Candida* species, it lacked activity against a significant proportion of *C. auris* isolates with mutations in *fks* target genes that conferred echinocandin cross-resistance. However, *fks1* mutations increased rezafungin MICs notably less than micafungin and anidulafungin MICs in *C. auris* [124]. Gathering all these results, rezafungin entered a Phase III multicenter, prospective, randomized, double-blind, efficacy and safety clinical trial in 2018 (ClinicalTrials.gov Identifier: NCT03667690) which is still currently recruiting for comparative evaluation of Rezafungin to caspofungin followed by optional oral fluconazole step-down therapy in subjects in subjects with candidemia and/or invasive candidiasis [125].

### 5.3. Selvamycin Analogues

**Figure 22:**
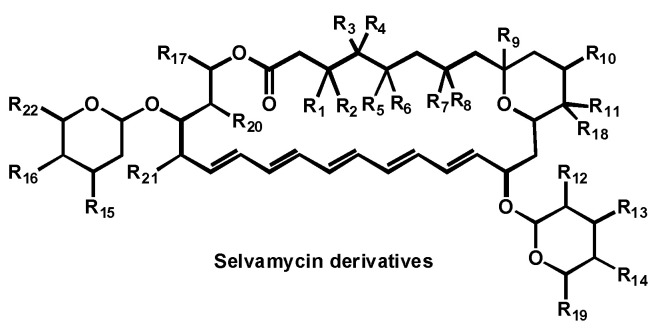
Structure of selvamycin derivatives.

In 2017, a patent, covering selvamicin and its analogues, was filed for the treatment of fungal infections (Figure 22) [126]. Selvamicin resembles the reference antifungals nystatin A1 and amphotericin B, but bears several distinctive structural features, impacting its pharmacokinetics and cell target. The data compiled in the patent showed that selvamicin and its analogues, compared to nystatin A1, are potent antifungal against *Candida* strains, including *C. auris*.

### 5.4. Polymers

Recently, Arias et al. reported the in vitro and in vivo activity of chitosan against planktonic and sessile forms of *C. auris*. In a *galleria mellonella* model of *C. auris* infection, chitosan decreased the killing effects of *C. auris* infection without any toxicity to the larvae. The putative mode of action of chitosan could be mediated by the expression of a stress-like gene expression response, conducting to protection in the larvae [127].

**Scheme 6:**
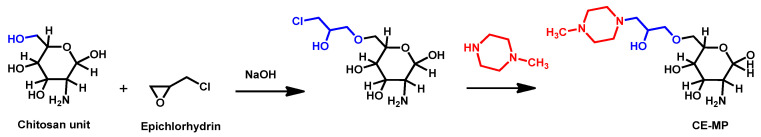
Synthesis of CE-MP as reported by Chauhan and Loonker.

However, in 2017, Chauhan and Loonker described the modification of chitosan to integrate *N*-methyl piperazine moieties and the biological evaluation of the resulting polymer called CE-MP [128]. They realized the cross-linkage of the naturally occurring linear polysaccharide matrix of chitosan with epichlorohydrin and then incorporated *N*-methyl piperazine moieties (Scheme 6). The resulting artificial polymer was fully characterized and proved to be active against some fungi but unfortunately displayed no activity against *C. auris*.

Taken together, these results demonstrated that naturally derivated polymers such as chitosan could be interesting alternatives to conventional antifungals.

### 5.5. Polyclonal Antibody

In 2018, Bujdakova et al. reported the activity of anti-CR3-RP polyclonal antibody against biofilms formed by *C. auris* [129]. During biofilm formation, *Candida* species generally expressed the complement receptor 3-related protein (CR3-RP) as surface antigens. The authors proved the presence of CR3-RP in *C. auris* cells within biofilms and the subsequent activity of anti-CR3-RP polyclonal antibody to eradicate the formation of biofilm by *C. auris*.

Polymers and polyclonal antibody could represent exciting therapeutic alternatives to small drugs but, for the moment, the number of works dealing with such strategies in the frame of fighting against *C. auris* is significantly restricted.

## 6. Traditional Medicines and Natural Compounds

It is notable that the reported data mainly resulted from in vitro evaluation. Few compounds experimented in vivo efficacy, unless specified.

### 6.1. Small Natural Compounds

**Figure 23:**
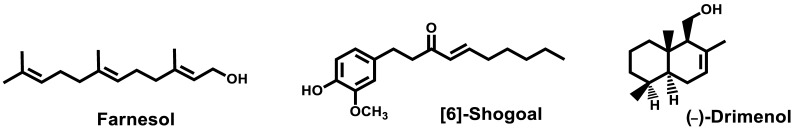
Structures of farnesol, [6]-shogoal and (−)-drimenol.

Farnesol, an endogenous quorum sensing molecule, was reported in 2020 by Vartika and Aijaz [130] for its ability to modulate development of biofilms and drug efflux in *C. auris*. Farnesol also blocked efflux pumps and downregulated biofilm and efflux pump associated genes at a concentration of 125 mM (Figure 23). In parallel, the effects of farnesol were also reported by Kovacs et al. [131,132]. The authors studied the effects of 100–300 µM farnesol on growth, biofilm production ability, production of enzymes related to oxidative stress, triazole susceptibility and virulence towards *C. auris* strains. After 24 h, farnesol was not able to inhibit the formation of biofilm but caused a significant growth inhibition against *C. auris* planktonic cells. Moreover, farnesol decreased the metabolic activity and increased the production of ROS. Used in combination with other antifungals, farnesol displayed a synergistic behavior. In vivo, using an immunocompromised murine model of disseminated candidiasis, daily 75 µM farnesol proved to lower the fungal burden and could be a benefit as therapeutics or adjuvant for the treatment of candidiasis.

[6]-Shogoal, one of the pungent constituents of ginger, exhibited antifungal and antibiofilm formation effects against *C. auris* [133]. The mode of action of [6]-shogoal was experimentally related to the reduction of the levels of aspartyl proteinases and downregulation of the expression of the efflux pump-related *CDR1*-gene in *C. auris*. However, the in vitro activity displayed by [6]-shogoal, even higher than fluconazole, is quite modest with MIC_80_ ranging from 32 to 64 µg/mL.

Eight drimane sesquiterpenoids including (−)-drimenol and (+)-albicanol were synthesized from (+)-sclareolide and evaluated for their antifungal activities. (−)-Drimenol (Figure 23) proved to limit the growth of *C. auris* better than fluconazole in the same concentration [134]. This natural compound also protected *C. elegans* from death in a candiasis model [134].

**Figure 24:**
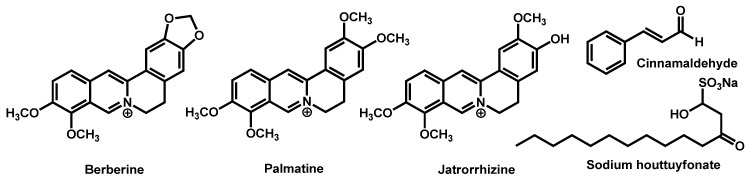
Composition of the five most common traditional herbal monomers as stated by Liu et al.

Traditional herbal monomers (THMs), mainly composed of sodium houttuyfonate (SH), berberine (BER), palmatine (PAL), jatrorrhizine (JAT), and cinnamaldehyde (CIN), were evaluated for their potential to induce cell wall modulations in *C. auris* (Figure 24) [135]. The authors proved that combination of these herbal monomers induced good antifungal activity against *C. auris* isolates the most potent combinations being SH/CIN and BER/PAL/JAT.

Bark and leaf essential oils from *Cinnamomum zeylanicum* proved in vitro antifungal activity against *C. auris*, by damaging the membrane and blocking the hyphae formation. From a chemical point of view, the main components of *C. zeylanicum* leaf is eugenol (62%) and *C. zeylanicum* bark is *trans*-cinnamaldehyde (66%), the other components being present at less than 7% [136].

Even if the use of natural compounds is of great interest, the activity of farnesol, [6]-shogoal, (−)-drimenol or common traditional herbal monomers is quite low compared to synthetic drugs and they have to be envisioned as adjuvants or platform molecules for drug chemical development.

### 6.2. Extracts of Natural Organisms

Marine sponges are among the richest sources of bioactive ingredients from marine organisms. In 2017, Hasaballah et al. reported the biological evaluation of crude extracts of marine sponges, *Negombata magnifica* and *Callyspongia siphonella* towards a large panel of biological targets, including *C. auris*. In addition to their high insecticidal capacity, the two sponge extracts also displayed high antibacterial and antifungal activities, with growth inhibition of 15.3 ± 1.2 mm for *N. magnifica* and 13.7 ± 1.5 mm for *C. siphonella* compared to 19.8 ± 0.63 mm for amphotericin B [137].

**Figure 25:**
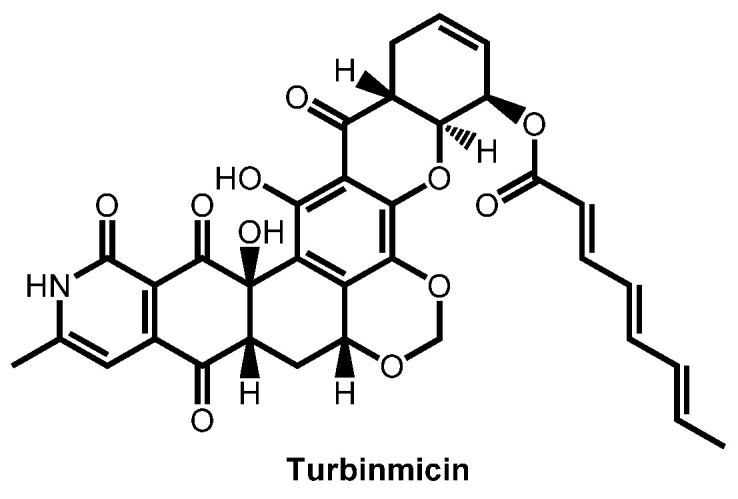
Structure of turbinmicin.

In 2020, Zhang et al. reported the identification of turbinmicin, a lead antifungal which was identified from marine microbiome (Figure 25). Turbinmicin is highly interesting because it functions through a fungal-specific mode of action, targeting Sec14 of the vesicular trafficking pathway [138]. Moreover, the same team reported the striking ability of turbinmicin to block biofilm formation. Indeed, turbinmicin acts by disruption of extracellular vesicle delivery during biofilm growth [139]. Turbinmicin revealed as a promising anti-biofilm and antifungal drug [140].

### 6.3. Peptides and Derivatives

In 2015, Cm-p5, an antifungal hydrophilic peptide derived from the coastal mollusk *cenchritis muricatus* was reported by Lopez-Abarrategi et al. [141]. Cm-p5 proved to have a fungistatic activity against *C. albicans* with MIC around 10 µg/mL and moderate toxicity toward mammalian cell lines. Following this first report, the same team obtained mutants of Cm-p5 and then designed and synthetized a helical-stabilized, cyclic, and nontoxic analogue of Cm-p5. This analogue displayed moderate antifungal activity against *C. auris* [142]. In 2020, Rosenau et al. also reported that the same derivatives of the Cm-p5 were able to inhibit the development of *C. auris* biofilms in vitro [143].

Ramachandran et al. evaluated the efficacy of three novel cyclic lipopeptides of the class *Bacillomycin* to limit fungal infection [144]. The lipopeptide analogues, bearing the same peptide sequence Asn-Pro-Tyr-Asn-Gln-Thr-Ser, were purified from a cell-free supernatant of *Bacillus subtilis* RLID 12.1. The three active lipopeptides exhibited MICs from 3.5 to 8.5 µg/mL against 10 *C. auris* isolates.

Antimicrobial peptides (AMPs) constitute a key component of these innate immune defences and AMPs from a variety of sources have shown potent antifungal activities [145,146,147,148]. Since they induce no or minimal MDR in target fungi, AMPs are considered strongly attractive as potential therapeutic drugs [149]. Pathirana et al. have investigated salivary Histatin 5 (Hst 5) for its ability to limit fungal infections [150]. Hst 5 is a well-studied salivary cationic peptide. After treatment at a 7.5 µM dose, 55 to 90% of all *C. auris* cells were killed by Hst 5, irrespective of the MDR-profile strains. The mode of action of Hst 5 is based on its translocation to the cytosol and vacuole and the reported study suggested that it differed from the one of fluconazole. Baso, Garcia et al. recently reported the antifungal properties of θ-defensins, macrocyclic peptides expressed in tissues of old world monkeys [151]. The mode of action of Rhesus θ-defensin 1 (RTD-1), the prototype θ-defensin, occurred by cell permeabilization, correlated with ATP release and intracellular accumulation of killing reactive oxygen species. Other natural defensins isoforms were evaluated and the most promising proved to be more active than caspofungin and/or amphotericin B caspofungin against fluconazole-resistant organisms, including *C. auris*. θ-defensin activity was compared with Hst 5 and proved to be 200-fold more active and more stable to proteases.

Dal Mas et al. described the effect of Crotamine, a natural antimicrobial peptide (AMP) isolated from a South American rattlesnake on *C. auris* [152]. Crotamine, which has structural similarities with human defensins, exhibited in vitro activity against most isolates tested, whereas these *Candida* isolates showed resistance to amphotericin B and fluconazole.

Cathelicidins are a class of epithelial antimicrobial peptides that are expressed in the intestinal epithelium and kill microorganisms by membrane disruption. Haagsman et al. recently obtained cathelicidin-inspired antimicrobial peptides and evaluated their antifungal capacity [153]. These novel antimicrobial peptides termed ‘PepBiotics’, containing between 21 and 37 residues, have been patented and showed a strong inhibitory activity against a large panel of fungi and yeasts at low concentrations (≤1 μM). In particular, they exhibited MIC = 0.6–1.3 µM against *C. auris* strains.

### 6.4. Bioinspiration

**Figure 26:**
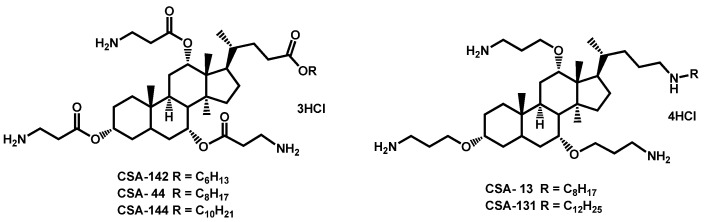
Structures of selected ceragenins CSAs.

With the goal to mimic AMPs, Savage et al. recently developed ceragenins (CSAs), a class of non-peptide molecules, based on a common bile acid, that conserve the amphiphilic morphology common to AMPs (Figure 26). They have then evaluated the susceptibilities of *C. auris* isolates, in biofilm and planktonic forms, to CSAs. In addition, they also measured the effectiveness of the most promising CSAs in gel and cream formulations in treated infected tissue explants [154]. Lead CSAs led to activity comparable with reference drugs and no cross-resistance was observed. In ex-vivo mucosal tissues, CSAs were able to significantly reduce the fungal burden with 2% topical application.

### 6.5. Necrotrophic Mycoparasitism

Necrotrophic mycoparasitism describes the ability of a fungal species to kill other fungi [155]. In 2018, Junker et al. examinated the use of a predatory yeast, *Saccharomycopsis schoenii*, as a potential biocontrol agent against *C. auris* [156]. They investigated the interaction between the two species by seeding equal quantity of dimorphic *S. schoenii* and ovoid *C. auris* NCPF8985#20 cells on minimal media agar on microscopy slides. Using specialized penetration pegs, *S. schoenii* attacked *C. auris* cells upon contact and killed 34% of them within a period of 6 h. All isolates of *Candida* species tested, including several drug resistant *C. auris* strains, were susceptible to predation by *S. schoenii*, opening new possibilities to eradicate such multidrug resistant strains.

## 7. Metal, Metal Complexes and Metalloids

Recently, Goldman et al. evaluated the potential of gallium, already known for its antibacterial activity, as antifungal [157]. They demonstrated that gallium nitrate had a fungistatic action against fungi. Regarding *C. auris*, several strains (473/2015, 490/2015, 501/2015, and 502/2015) were completely inhibited by gallium at concentrations of 128–256 mg/L. However, others (strains 467/2015, 470/2015, and 484/2015) survived the challenge at the higher concentration tested limiting the use of gallium as a broad-spectrum antifungal agent.

**Figure 27:**
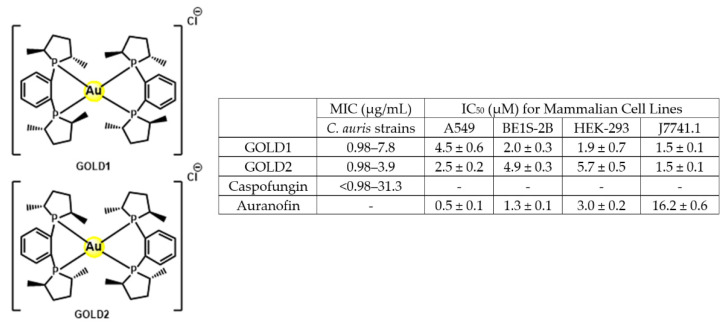
Gol(I)-complexes as antifungals: structure, MIC values range and cytotoxicity.

In 2020, the team of Garneau-Tsodikova and Awuah explored the potential of linear and square-planar gold(I)−phosphine complexes as antimicrobial agents against a panel of 28 fungal strains [158]. Compounds GOLD1 and GOLD2 proved to be the most efficient against *C. auris*, with MIC similar to caspofungin. Moreover, these two gold-complexes displayed low cytotoxicity against a panel of cell lines (Figure 27).

The amount of data gathered on the use of metal and metallic complexes towards *C. auris* is too low to ascertain the interest of such strategy, but the first data are encouraging. Therefore, medicinal chemists should go further with this alternative area of research in the fight against fungal resistant strains.

## 8. Others Approaches

### 8.1. Probiotics and Postbiotics

The review by Zhang et al. described the recent reports that indicate that **probiotics** may also contribute to protect against fungal infections [159]. Even if some publications have evaluated the benefits of probiotics against fungal infections caused by *C. albicans*, *C. glabrata*, *A. fumigatus*, *T. tonsurans*, *M. canis*, *M. gypseum*, or *E. flocossum*, no reports on *C. auris* are presented in that review published in 2019.

Late 2019, Rao et al. exploited the probiotic properties of two novel, food-derived yeasts, *Saccharomyces cerevisiae* (strain KTP) and *Issatchenkia occidentalis* (strain ApC). These alternative approaches to combat widespread opportunistic fungal infections proved to be effective to inhibit virulence traits such as adhesion, biofilm formation and filamentation of Candida spp., especially *C. auris* [160,161].

In 2020, Campos-Junqueira et al. described the pro- and postbiotic activity of *Lactobacillus paracasei* 28.4 against *C. auris*. It is notable that they conducted an in vivo study with *G. mellonella* larvae infected with *C. auris*. Injections of LPCE and LPF1 (crude extract and fraction 1 derived from *L. paracasei* 28.4 supernatant, respectively) prolonged survival of these insects compared to a control group (*p* < 0.05) and modulated the host immune response [162].

### 8.2. Nanoparticles and Coatings

In 2019, Philip and Kuriakose reported the synthesis of superparamagnetic Fe_2_O_3_ nanoparticles stabilized by biocompatible supramolecular β-cyclodextrin and their evaluation against three strains of *C. auris* but with a moderate MIC = 500 µg/mL [163].

Chapman, Truong et al. reported in 2020 the fabrication of long-term microbicidal silver nanoparticle clusters [164]. This silver nanoparticle cluster coating was constructed on copper surface using an ion-exchange and reduction reaction. The resulting surface was contaminated by *C. auris* and evaluated at extended periods. After seven days, 90% of *C. auris* proved to be non-viable on the new designed surface.

In parallel, using a microwave assisted synthetic approach, Lara et al. reported the synthesis of pure round silver nanoparticles (AgNPs) and their use to inhibit *C. auris* biofilm formation on surfaces [165]. These AgNP-treated biofilms showed cell wall damage mostly by disruption and distortion of the outer surface of the fungal cell wall. Moreover, the AgNPs-functionalized fibers proved to be stable and kept their fungicidal potential even after repeated thorough washes.

Very recently, Nosanchuck et al. described the effects of nanoparticles capable of generating nitric oxide (NO) to kill *C. auris* [166]. This NO-nanotherapeutics were incubated with six different *C. auris* strains and eradicated both planktonic and biofilm *C. auris* with a 10 mg/mL and emerged as a promising approach to fight against MDR-fungal strains.

Bismuth nanoparticles (BiNPs) demonstrated strong antifungal activity against a panel of *C. auris* strains, with MIC ranging from 1 to 4 µg/mL. Moderate effects of these nanoantibiotics were also examined by scanning electron microscopy and showed the disruption of the *C. auris* cell morphology and the biofilm structure [167].

### 8.3. Hydrogels

In 2019, Shukla and Vera-Gonzalez patented an aspartic protease-triggered antifungal hydrogel able to locally deliver antifungal drugs that specifically respond to aspartic proteases secreted by virulent and pathogenic *Candida* species [168].

In 2020, Rosenau et al. described a novel anti-infective multicomponent gatekeeper hydrogel [169]. This bilayer hydrogel protected the patient from invasion by *C. auris* from infected wounds, by combining to complementary layers. The first layer reduced the loading of the pathogen *C. auris* by selective cell capturing within the affinity layer of the gel followed by subsequent inactivation within the therapeutic gel layer.

In a similar manner, Gupta et al. reported similar antibacterial silver-nanoparticle hydrogel for wound dressing applications [170]. The particularity of their design relied on the green chemistry approach—based on natural curcumin as reducing agent-developed to obtain the nanoparticles.

### 8.4. Irradiation

Candida spp. are the most common cause of fungal infections worldwide and the fifth most common cause of nosocomial infections [171]. In particular, *C. auris* resists disinfection with cleaning agents widely used in hospitals and long-term care facilities [31,172], is frequently drug resistant, and can persist in the environment for months [173]. As a consequence, new protocols to identify how to eliminate *C. auris* from surface and devices drove several studies.

In 2018, Cadnum et al. stated about the relative resistance of *C. auris* to be eradicated by ultraviolet light [174]. Before this study, it was not clear if mobile ultraviolet-C (UV-C) light room decontamination devices efficiently cleaned healthcare facilities. From their data, it appeared that *C. auris* was significantly less susceptible to killing by UV-C than methicillin-resistant *Staphylococcus aureus*. This first statement brought to light the need in understanding how efficiently eradicate *C. auris* using UV lights. Consequently, in 2019, Ponnanchan et al. evaluated the antifungal activity of ultraviolet C (UVC) light using mercury vapour lamp with a peak emission of 254 ± 2 nm against 32 *C. auris* isolates. Contaminated plates were held parallel to the UVC source throughout the period of exposure. After 15 min of irradiation at this wavelength, all experiments demonstrated complete inhibition of growth up to 72 h incubation for *C. auris* [35].

In a parallel manner, in 2019, Maslo et al. reported the efficacy of pulsed-xenon ultraviolet light to eradicate *C. auris* [175]. After inoculation, each sample was exposed during uninterrupted periods of 5, 10 or 15 min at distances ranging from 1 to 2 m. The PX-UV mobile device killed 99.4% of *C. auris* CFU on the surface after 5 min irradiation at 1 m-distance. The time and distance of exposure have also been quantified by de Groot et al. [176]. The optimum for *C. auris* eradication was obtained after 30 min of decontamination at 2 m. Dividing time by two or double the distance decreased the activity by 10- and 50-fold, respectively.

In 2020, Lemons et al. also demonstrated the efficiency of ultraviolet irradiation to kill *C. auris* [177]. Ultraviolet germicidal irradiation (UVGI) was investigated to inactivate ten clinically relevant strains of *C. auris*. In order to determine dose-response curves, each sample was exposed eleven times to range of UV-dose from 10 to 150 mJ/cm^2^. From these data, *C. auris* required more energy than *C. albicans* to be eradicated. As a consequence, the authors stated that UVGI could be applied against *C. auris* but the variation of susceptibility would have to be considered.

From these reports, irradiation could be a powerful tool to eradicate *C. auris* in medical area. However, all parameters should be carefully estimated to avoid a lack of effectiveness and propose tailor-made solutions to eradicate *C. auris*.

## 9. Conclusions and Perspectives

Through gathering all these data it appeared that the fight against *C. auris* is continuously increasing for the last five years, in parallel of its emergence and definition as a fungus of concern. Even if most reports dealt with *Candida* species, some important works focused only on *C. auris* and took in consideration its own particularities. No perfect solution has been stated for the moment, but clear opportunities have been demonstrated whether from drug repositioning, combination of drugs, or the design of novel antifungal entities. From a medicinal chemist point of view, some molecular features seemed to emerge as required for the design of potent antifungals against *C. auris*, and there is no doubt that these preferred moieties should inspire the conception of novel effective drugs. As always, nature is a great provider of active compounds and bioinspiration also drives some exciting research works. Even if it is of great importance to treat patients, it is also absolutely required to clean devices and surfaces to avoid contamination and proliferation. Therefore, the emerging solutions based on the specific characteristic of *C. auris* in terms of irradiation or antimicrobial surfaces are of particular interest. Such an approach could be considered as tailor-made conception towards *C. auris*. To summarize, ongoing works are very encouraging, but there is still an urgent need to propose therapeutic alternatives to eradicate *C. auris*.

## Figures and Tables

**Table 1:** Active repositionable drugs against *C. auris* according to Zagaroza et al.

Structure	Name	Class	Growth Inhibition (%) at 50 µM		
			CL 10093	JCM 15448	KCTC 17810
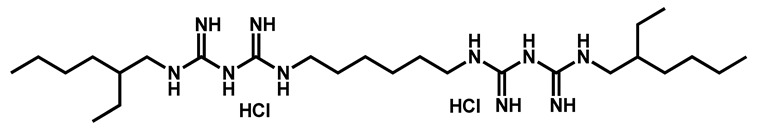 Alexidine dihydrochloride		Antibacterial	97	97	97
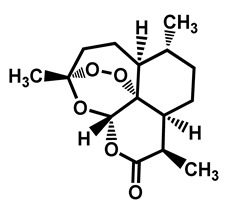	Artemisinin	Antimalarial	65	56	61
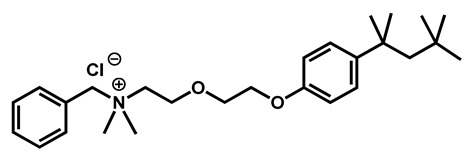 Benzethonium chloride		Antibacterial	96	97	100
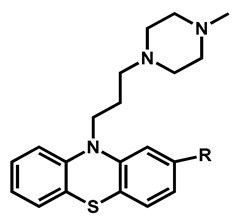 Chlorhexidine		Antibacterial	98	99	98
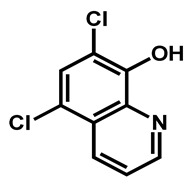	Chloroxine	Antibacterial	95	97	98
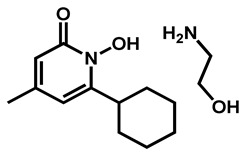	Ciclopirox ethanolamine	Antibacterial/Antifungal	94	97	94
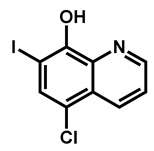	Clioquinol	Antiamebic/Antibacteria	89	93	93
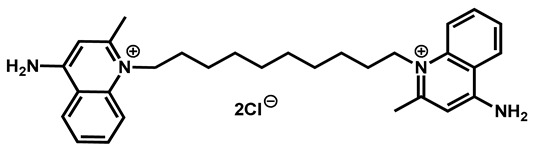 Dequalinium dichloride		Antibacterial	81	86	89
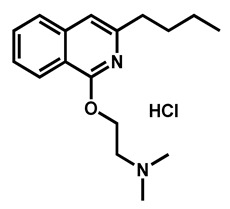	Dimethisoquin hydrochloride	Antipruritic	59	65	51
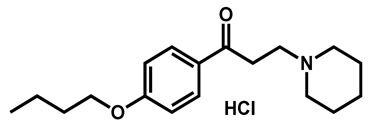	Dyclonine hydrochloride	Local anesthetic	63	63	54
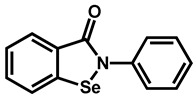	Ebselen	Anti-inflammatory	87	91	92
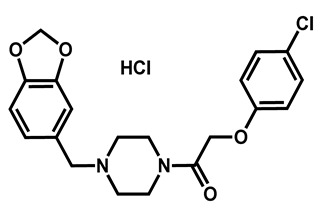	Fipexide hydrochloride	Anti-fatigue	68	63	54
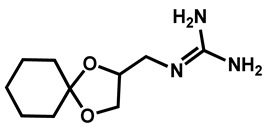	Guanadrel sulfate	Antihypertensive	97	97	97
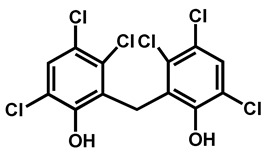	Hexachlorophene	Antiseptic	97	98	86
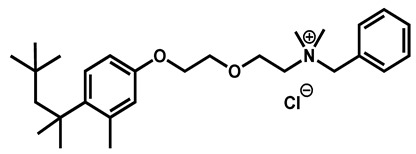	Methyl benzethonium chloride	Antibacterial	98	97	100
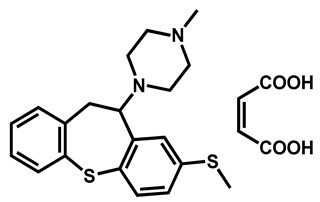	Methiothepin maleate	Antipsychotic	74	72	64
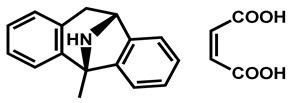	MK 801 hydrogen maleate	Anticonvulsant	98	98	97
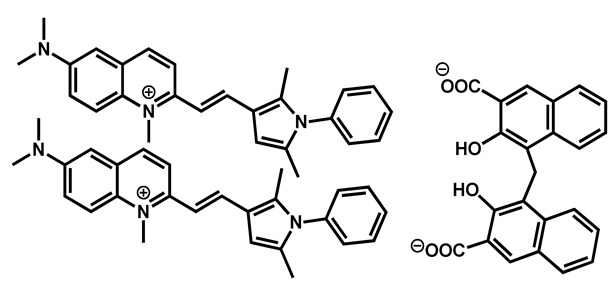 Pyrvinium pamoate		Anthelmintic	40	76	61
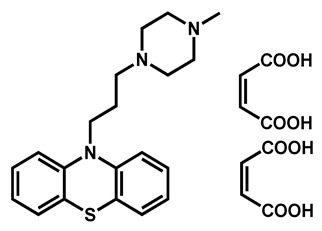	Prochlorperazine dimaleate	Antiemetic/Antipsychotic	71	66	72
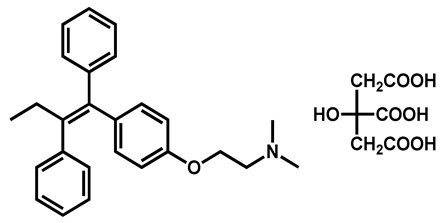	Tamoxifen citrate	Antineoplastic	98	98	90
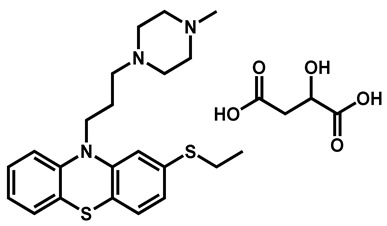	Thiethylperazine dimalate	Antiemetic	68	90	86
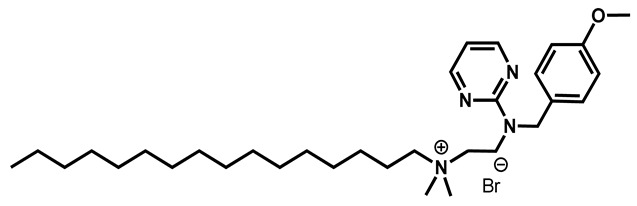 Thonzonium bromide		Antiseptic	98	98	98
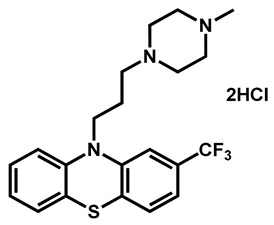	Trifluoperazine dihydrochloride	Antiemetic	54	88	61
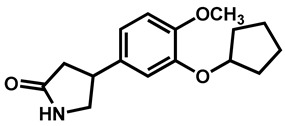	Rolipram	Antidepressant	98	97	91
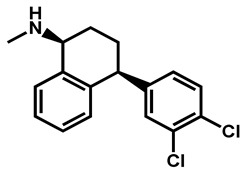	Sertraline	Antidepressant	59	88	56
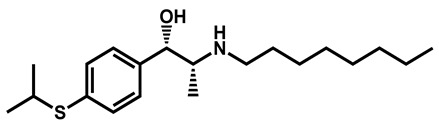	Suloctidil	Antiplatelet	96	99	78
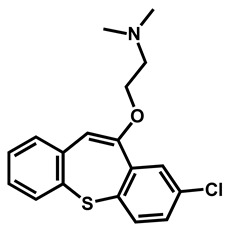	Zotepine	Antipsychotic	59	63	56

**Table 2:** Critical analysis of quinoline/isoquinoline repositionable drugs.

Entry	Structures	Log *P* ^1^	TPSA ^2^ (Å)	M (g/mol)	Druglikeliness ^3^ (Alert)	BBB Permeant ^4^	GI Absorption ^5^
1	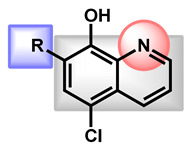 R = I = Clioquinol R = Cl = Chloroxine	2.96	33.12	305.50	XLOGP > 3.5	Yes	High
2		2.86	33.12	214.05	MW < 250, XLOGP > 3.5	Yes	High
3	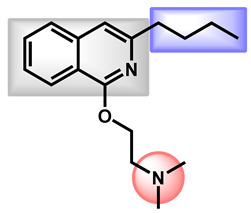 Dimethisoquin	3.66	25.36	272.39	XLOGP > 3.5	Yes	High
4	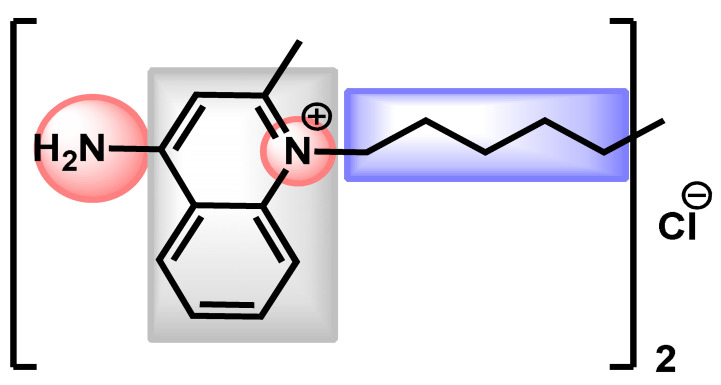 Dequalinium dichloride	4.14	59.80	456.67	MW > 350 Rotors > 7 XLOGP > 3.5	No	High
5	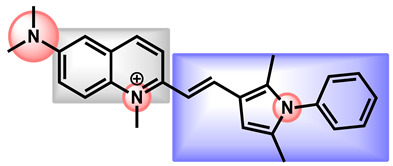 Pyrvinium active compound	4.29	12.05	382.52	MW > 350 XLOGP > 3.5	Yes	High

^1.^ Log*P* = octanol/water partition coefficient calculated by SwissADME; ^2^ TPSA = topology polar surface area by SwissADME; ^3^ Druglikliness = criteria which should be problematic for drug development according to SwissADME; ^4^ BBB permeant: blood brain barrier permeant as predicted by SwissADME; ^5^ gastro-intestinal permeation ability as predicted by SwissADME.

**Table 3:** Critical analysis of benzothiepine/phenothiazine repositionable drugs.

Entry	Structure	R	Log*P* ^1^	TPSA (Å) ^2^	M (g/mol)	Druglikeliness (Alert) ^3^
1	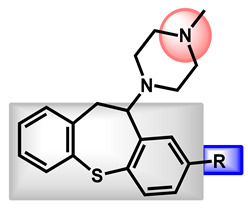	-S-CH_3_ (Methiothepin)	3.94	57.08	356.55	MW > 350, XLOGP > 3.5
2		-Cl (Zotepine)	4.37	37.77	331.86	XLOGP > 3.5
3	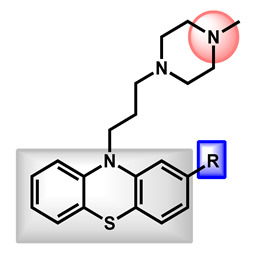	-H (Prochlorperazine)	3.47	35.02	339.50	XLOGP > 3.5
4		-S-CH_2_CH_3_ (Thiethylperazine)	4.43	60.32	399.62	MW > 350, XLOGP > 3.5
5		-CF_3_ (Trifluoperazine)	4.53	35.02	407.50	MW > 350, XLOGP > 3.5

^1.^ Log*P* = octanol/water partition coefficient calculated by SwissADME; ^2^ TPSA = topology polar surface area by SwissADME; ^3^ Druglikliness = criteria which should be problematic for drug development according to SwissADME.

**Table 4:** Critical analysis of **amide-based** repositionable drugs.

Entry	Structures	Log*P* ^1^	TPSA ^2^ (Å)	M (g/mol)	Druglikeliness ^3^ (Alert)	BBB Permeant ^4^	GI ^5^ Absorption
1	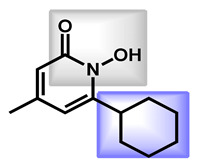 Ciclopirox	2.38	42.23	207.27	MW < 250	Yes	High
2	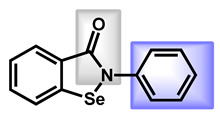 Ebselen	1.75	22.0	274.18	No alert	Yes	High
3	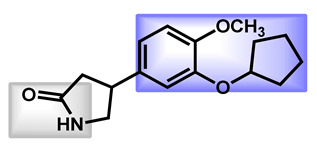 Rolipram	2.44	47.56	275.34	No alert	Yes	High
4	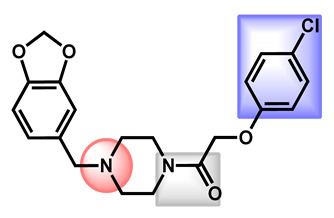 Fipexide	2.82	51.24	388.84	MW > 350	Yes	High
5	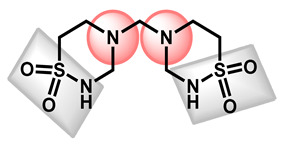 Taurolidine	−1.53	115.58	284.36	0 alert	No	Low

^1.^ Log*P* = octanol/water partition coefficient calculated by SwissADME; ^2^ TPSA = topology polar surface area by SwissADME; ^3^ Druglikliness = criteria which should be problematic for drug development according to SwissADME; ^4^ BBB permeant: blood brain barrier permeant as predicted by SwissADME; ^5^ gastro-intestinal permeation ability as predicted by SwissADME.

**Table 5:** Antifungal activity of phenolic compounds against *C. auris* as reported by Ahmad et al.

	Test Agents	Structure	MIC Values (µg/mL) (*n* = 3)
			Median (Range)
*C. auris*	Carvacrol	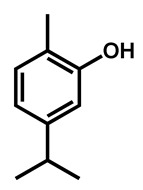	125 (62–250)
	Thymol	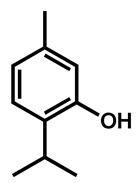	312 (156–625)
	Eugenol	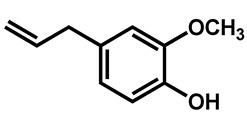	625 (312–1250)
	Methyl eugenol	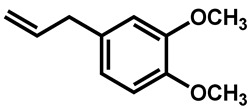	1250 (625–1250)

**Table 6:** Antifungal activities of compound BM1 against various *Candida* species.

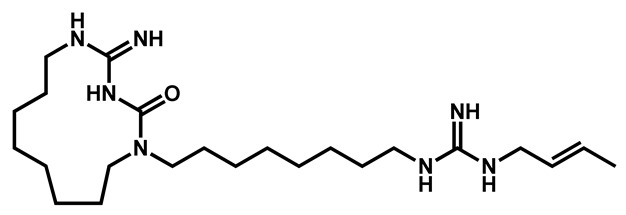 BM1											
MIC and MBC Rage (µg/mL) against *Candida* Species											
*C. albicans*		*C. tropicalis*		*C. parapsilosis*		*C. glabrata*		*C. krusei*		*C. auris*	
MIC	MBC	MIC	MBC	MIC	MBC	MIC	MBC	MIC	MBC	MIC	MBC
0.125–2	2–4	2	2–4	8–16	32–64	32–64	8–64	8–64	64–128	8–64	128–256

MBC, minimum bactericidal concentration; MIC, minimum inhibitory concentration.

## Data Availability

Not applicable.
